# An updated systematic review and meta-analysis on adherence to mediterranean diet and risk of cancer

**DOI:** 10.1007/s00394-020-02346-6

**Published:** 2020-08-08

**Authors:** Jakub Morze, Anna Danielewicz, Katarzyna Przybyłowicz, Hongmei Zeng, Georg Hoffmann, Lukas Schwingshackl

**Affiliations:** 1 Department of Cardiology and Internal Diseases, University of Warmia and Mazury, al. Warszawska 30, 10-082 Olsztyn, Poland; 2 Department of Human Nutrition, University of Warmia and Mazury, ul. Sloneczna 45f, 10-718 Olsztyn, Poland; 3 National Cancer Registry Office, National Cancer Center, 17 South Lane, Beijing, 100021 China; 4 Department of Nutrition, Harvard T.H. Chan School of Public Health, 655 Huntington Ave, Building 2, Boston, MA 02551 USA; 5 Department of Nutritional Sciences, University of Vienna, Althanstraße 14, UZA II, 1090 Vienna, Austria; 6 Institute for Evidence in Medicine, Medical Center-University of Freiburg, Faculty of Medicine, University of Freiburg, Breisacher Straße 153, 79110 Freiburg, Germany

**Keywords:** Mediterranean diet, Cancer, Meta-analysis, Certainty of evidence

## Abstract

**Purpose:**

The aim of current systematic review was to update the body of evidence on associations between adherence to the Mediterranean diet (MedDiet) and risk of cancer mortality, site-specific cancer in the general population; all-cause, and cancer mortality as well as cancer reoccurrence among cancer survivors.

**Methods:**

A literature search for randomized controlled trials (RCTs), case–control and cohort studies published up to April 2020 was performed using PubMed and Scopus. Study-specific risk estimates for the highest versus lowest adherence to the MedDiet category were pooled using random-effects meta-analyses. Certainty of evidence from cohort studies and RCTs was evaluated using the NutriGrade scoring system.

**Results:**

The updated search revealed 44 studies not identified in the previous review. Altogether, 117 studies including 3,202,496 participants were enclosed for meta-analysis. The highest adherence to MedDiet was inversely associated with cancer mortality (RR_cohort_: 0.87, 95% CI 0.82, 0.92; *N* = 18 studies), all-cause mortality among cancer survivors (RR_cohort_: 0.75, 95% CI 0.66, 0.86; *N* = 8), breast (RR_observational_: 0.94, 95% CI 0.90, 0.97; *N* = 23), colorectal (RR_observational_: 0.83, 95% CI 0.76, 0.90; *N* = 17), head and neck (RR_observational_: 0.56, 95% CI 0.44, 0.72; *N* = 9), respiratory (RR_cohort_: 0.84, 95% CI 0.76, 0.94; *N* = 5), gastric (RR_observational_: 0.70, 95% CI 0.61, 0.80; *N* = 7), bladder (RR_observational_: 0.87, 95% CI 0.76, 0.98; *N* = 4), and liver cancer (RR_observational_: 0.64, 95% CI 0.54, 0.75; *N* = 4). Adhering to MedDiet did not modify risk of blood, esophageal, pancreatic and prostate cancer risk.

**Conclusion:**

In conclusion, our results suggest that highest adherence to the MedDiet was related to lower risk of cancer mortality in the general population, and all-cause mortality among cancer survivors as well as colorectal, head and neck, respiratory, gastric, liver and bladder cancer risks. Moderate certainty of evidence from cohort studies suggest an inverse association for cancer mortality and colorectal cancer, but most of the comparisons were rated as low or very low certainty of evidence.

**Electronic supplementary material:**

The online version of this article (10.1007/s00394-020-02346-6) contains supplementary material, which is available to authorized users.

## Introduction

Cancer is widely recognized as one of the leading public health issues worldwide. According to the GLOBOCAN estimates, in 2018 there were 18.1 million new cases of cancer and it contributed to the death of 9.6 million people [1]. Despite a decreasing trend in cancer mortality observed in recent years, it is still the second most common cause of death world-wide, second to cardiovascular diseases (CVD) [2]. Taking into account trends of recent years, e.g., in Europe and the US, there seems to be a transition concerning the distribution of these two main causes of death. It is reasonable to speculate that cancer will replace CVD as the major cause of death in years to come. According to recent data provided by the PURE study group, this has already happened in a number of high- as well as middle-income countries in adults aged 35–70 years [2]. Until 2040, the global burden of neoplasms is going to rise by more than half [3]. Due to simultaneous improvements in diagnosis and treatment approaches, there will be a substantial increase in the number of cancer survivors as well [4].

Irrespective of site-specific details in the pathogenesis of tumors, up to more than 90% of cancers are considered to be attributable to modifiable risk factors such as tobacco smoking, excessive body weight, physical inactivity, alcohol consumption, infectious agents, environmental pollution, and suboptimal diet [5, 6]. The latter is made responsible for about 5–10% of total cancer cases [5, 7, 8].

According to the World Cancer Research Fund (WCRF), high consumption of fruits, vegetables and whole grains, as well as low intake of red and processed meat can lower cancer risk. As food items and nutrients are consumed in combination, dietary patterns have been successfully implemented as a tool to assess the additive or synergistic effect of food in nutritional epidemiology [9, 10].

With regard to prevention of non-communicable diseases, one of the most well-represented dietary patterns in literature is the Mediterranean diet (MedDiet) [11]. The MedDiet is a plant-based pattern characterized by high amounts of fruits, vegetables, nuts, legumes, fish, cereals including whole grains, and extra-virgin olive oil, at the same time reducing intake of red, processed meat, eggs and dairy [12]. An additional component is a moderate intake of red wine [12]. A large body of clinical and epidemiological studies have observed the protective effect of the MedDiet on cardiovascular disease, diabetes, obesity as well as cancer [13].

We previously conducted a systematic review and meta-analysis on the association between adherence to the MedDiet and risk of cancer, which was followed by two updates [14–16]. In the last update, we were able to pool data from 83 studies (including randomized controlled trials, cohort and case–control studies) showing an inverse association between the highest MedDiet adherence category and the risk of cancer mortality as well as incidence of breast, colorectal, gastric, liver, head and neck, and prostate cancer [16]. Although little time has passed, since then, we decided to synthesize the available data in another update due to the following reasons. Since the publication of the latest version of the review, the number of new reports from cohort and case–control studies has increased substantially [17–19]. Additionally, some of the new studies focus on cancer subtypes not previously included in our reports [20, 21]. Moreover, we wanted to expand our findings by assessment of the certainty of evidence, which is rarely evaluated in nutrition research evidence syntheses.

Therefore, the aim of this review was to enhance our previous findings on adherence to the MedDiet pattern and risk of cancer mortality, site-specific cancer and all-cause as well as cancer mortality among cancer survivors. Additionally, we aimed to assess the certainty of evidence for identified comparisons.

## Methods

The protocol for previous versions of this review was published in PROSPERO International Prospective Register of Systematic Reviews (CRD42013004382). This update of the systematic review was planned and conducted according to the standards of the Preferred Reporting Items for Systematic Reviews and Meta-Analyses (PRISMA) statement [22].

### Search strategy

Two electronic databases, PubMed (from August 2017 to April 2020) and Scopus (from January 2017 to April 2020), were searched with no limitations to publication language. Following search terms were adopted for PubMed and Scopus: (“Mediterranean diet” OR “Mediterranean” OR “diet” OR “dietary pattern” OR “dietary score” OR “dietary adherence”) AND (“cancer” OR “neoplasm” OR “neoplastic disease” OR “survivors” OR “recurrence”) AND (“prospective” OR “follow-up” OR “cohort” OR “longitudinal” OR “case–control”). References from identified articles, systematic reviews and meta-analyses were screened for potential eligibility.

### Study selection

Two reviewers (J.M. and A.D.) independently evaluated the eligibility of studies with any disagreements resolved by discussion with the third reviewer (L.S.). In contrast to previous versions of this review, we expanded our analyses with all-cause mortality among cancer survivors. Studies were included if they fulfilled the following criteria: (1) randomized controlled trials (RCTs), prospective cohort, case–cohort, nested case–control, or case–control studies, (2) conducted in adult population (aged ≥ 18 years) which (3) assessed association between adherence to MedDiet and (4) risk of cancer mortality, site-specific cancer, all-cause, cancer mortality or cancer reoccurrence among cancer survivors. If several reports from a single study were available, the one with longer follow-up or a larger number of participants/cases was selected.

### Data extraction

After completing selection of eligible studies, two reviewers (J.M. and L.S.) extracted the following data: (1) name of first author, (2) country, (3) study name, (4) study design, (5) outcome, (6) population size, (7) number of cases, (8) length of the study follow-up, (9) age at entry, (10) sex, (11) composition of the MedDiet score and its range, (12) adjustment set and (13) multivariable risk estimates (odds ratio (OR), risk ratio (RR) or hazard ratio (HR) comparing groups of highest and lowest adherence to MedDiet) with corresponding 95% confidence intervals (CI). If a study presented several risk estimates, the one with maximal adjustment was chosen. If separate results for men and women or different cancer subtypes were presented in a study, the estimates were pooled using a fixed-effects model.

### Certainty of evidence assessment

To evaluate the certainty of evidence for associations between adherence to MedDiet and cancer outcomes in cohort studies and RCTs, the NutriGrade tool was adopted [23]. This tool is based on the following 9 items: (1) risk of bias, study quality, and study limitations (maximum 2 points for cohorts or 3 points for RCTs); (2) precision (maximum 1 point); (3) heterogeneity (maximum 1 point); (4) directness (maximum 1 point); (5) publication bias (maximum 1 point); (6) funding bias (maximum 1 point); (7) study design (+ 2 points—only for RCTs); (8) effect size (maximum 2 points—only for cohort studies); and (9) dose–response (maximum 1 point—only for cohort studies). Risk of bias domain was assessed using a checklist created by authors of the tool. Four categories based on the total score were used to interpret the certainty of evidence: very low (0 to < 4 points), low (4 to < 6 points), moderate (6 to < 8 points) and high (≥ 8 points).

### Statistical analysis

The meta-analysis was conducted by pooling the multivariable-adjusted RRs, HRs or ORs of the highest compared with the lowest MedDiet adherence category using a random-effects model with the DerSimonian–Laird method [24]. Outcomes in the meta-analysis were assumed to be ORs in case-control studies and  RRs in prospective studies and RCTs. Using an inverse variance method, the standard error (SE) for the log-transformed OR/RR was calculated and interpreted as an estimated variance of log-transformed OR/RR to weight each study [24]. Included studies were categorized according to the following clinical outcomes: (1) cancer mortality, (2) biliary tract cancer, (3) bladder cancer, (4) blood cancer, (5) breast cancer, (6) colorectal cancer, (7) endometrial cancer, (8) esophageal cancer, (9) gallbladder cancer, (10) gastric cancer, (11) glioma, (12) head and neck cancer, (13) liver cancer, (14) ovarian cancer, (15) pancreatic cancer, (16) prostate cancer, (17) respiratory cancer, (18) skin cancer, (19) all-cause mortality, (20) cancer mortality, and (21) cancer reoccurrence among cancer survivors. Estimates from case–control, cohort studies and RCTs were compared separately. Joint estimates for observational studies were obtained by pooling together data from case–control and cohort studies in the same model. Additional analyses were conducted for associations between individual components of the MedDiet and overall cancer risk:

- Alcohol (within the range vs. higher consumption)
- Cereals (higher vs. lower consumption)
- Dairy (lower vs. higher consumption)
- Fish (higher vs. lower consumption)
- Fruit (higher vs. lower consumption)
- Legumes (higher vs. lower consumption)
- Meat (lower vs. higher consumption)
- Nuts (higher vs. lower consumption)
- Olive oil (higher vs. lower consumption)
- Vegetables (higher vs. lower consumption)
- Whole grains (higher vs. lower consumption)

*I*^2^ statistic and Cochran’s *Q* test were used to evaluate the heterogeneity between studies. For the *I*^2^ value greater than 50% indicated a substantial statistical heterogeneity [25]. Subgroup analyses were conducted only for prospective cohort studies, for comparisons which included ≥ 10 studies and were stratified for sex (male/female), geographical location (Mediterranean/non-Mediterranean countries) and type of MedDiet score (Trichopoulou MedDiet score [12]/Fung MedDiet score [26]). For breast cancer, pooled risk estimates were additionally compared by menopausal status (premenopausal/postmenopausal) and receptor expression (ER/PR/HER/mixed). Furthermore, analysis for colorectal cancer risk was run separately for anatomical location (proximal colon/distal colon/rectum).

For comparisons with ≥ 10 studies, small-study effects, such as publication bias, were explored by funnel plots and Egger’s regression test, as recommended by Cochrane Collaboration [27]. All analyses were conducted in Review Manager version 5.3 (Nordic Cochrane Center, Copenhagen, Denmark) and R version 3.6.1 (R Foundation for Statistical Computing, Vienna, Austria) with the “metafor” package [28].

## Results

### Database search and study characteristics

The updated literature search revealed 3720 publications after removal of duplicates from different databases. Additionally, 83 studies identified in previous versions of this systematic review were re-considered [29–111]. After title-abstract screening, 137 articles were assessed for eligibility and 20 articles were excluded at this step (ESM Table 1). Details of the study search and selection process were presented as a PRISMA-compliant flowchart in ESM Fig. 1.

Main characteristics of studies identified in the updated search are summarized in Table 1. Overall, 117 studies (with 12 case–control [112–123], 26 cohort [17–21, 124–144], five case–cohort [145–149], and one RCT (corrected report) [111] not identified in previous versions of this review) pooling 3,202,496 participants were included in the update [17–21, 29–34, 36–63, 65–72, 80–149].

**Table 1:** General characteristics of newly added case–control, case-cohort, cohort studies and randomized controlled trials identified in the updated literature search

Author (year)	Country Study name	Study design	Outcome	Population Follow-up (years)	Age at entry (years)	Sex	Components of score	Adjustment	OR/RR/HR (95% CI) Multivariable adjusted
Toledo et al. (2018)	Spain PREDIMED	RCT	Breast cancer	4152 4.8 years	60–80	W	1. MD supplemented with extra-virgin olive oil; 2. MD supplemented with mixed nuts; 3. control diet (advice to reduce dietary fat);	Age, BMI, waist to hip ratio, use of hormone therapy, leisure-time physical activity, total energy intake, alcohol consumption, age at menopause, baseline adherence to MD, propensity score, recruitment center, educational level	MD with olive oil RR 0.31 (0.13, 0.77) versus control diet MD with nuts RR 0.53 (0.23, 1.26) versus control diet Both MD RR 0.41 (0.19, 0.86) versus control diet
Boden et al. (2019)	Sweden VIP	Prospective cohort	Colorectal cancer Lung cancer Pancreatic cancer Gastric cancer Prostate cancer Breast cancer	100,881 15.0 years	30–60	M/W	aMDS score range: 0–8. 1.↑ vegetables and potatoes; 2.↑ fruit and fresh juices; 3.↑ fish and fish products; 4.↑ MUFA + PUFA:SFA ratio; 5.↑ whole-grain cereals; 6.↔ alcohol; 7.↓ meat and meat; 8.↓ dairy products;	Energy intake, BMI, physical activity, smoking, educational status	Colorectal cancer HR 1.02 (0.94, 1.10) Lung cancer HR 0.90 (0.80, 1.01) Pancreatic cancer HR 0.90 (0.76, 1.07) Gastric cancer HR 0.85 (0.69, 1.03) Prostate cancer HR 0.98 (0.92, 1.03) Breast cancer HR 0.98 (0.92, 1.05) Per one tertile increase
Bogumil et al. (2019)	USA MEC	Prospective cohort	Hepatocellular cancer	169,806 17.0 years	45–75	M/W	aMED score range: 0–9. 1.↑ vegetables; 2.↑ fruits; 3.↑ nuts; 4.↑ legumes; 5.↑ fish; 6.↑ whole grains; 7.↑ MUFA:SFA ratio; 8.↓ red and processed meat; 9.↔ alcohol;	Age, sex, race/ethnicity, BMI, history of diabetes, smoking status, energy intake	Hepatocellular cancer HR 0.68 (0.51, 0.90) for fifth versus first quintile
Bonaccio et al. (2018)	Italy MS	Prospective cohort	Cancer mortality	5200 8.1 years	≥ 35	M/W	MDS score range: 0–9. 1.↑ vegetables; 2.↑ legumes; 3.↑ fruits and nuts; 4.↑ cereals; 5.↑ fish; 6.↑ MUFA:SFA ratio; 7.↓ meat, 8.↓ dairy products; 9.↔ alcohol;	Sex, age, education, household income, leisure-time physical activity, smoking status, BMI, cancer, CVD, diabetes, hypertension, hypercholesterolemia, use of anti-depressants and energy intake	Cancer mortality HR 0.87 (0.57, 1.32) for third versus first tertile
Dela Cruz et al. (2020)	USA MEC	Prospective cohort	Breast cancer	101,291 17.4 years	45–75	W	aMED score range: 0–9. 1.↑ vegetables; 2.↑ fruits; 3.↑ nuts; 4.↑ legumes; 5.↑ fish; 6.↑ whole grains; 7.↑ MUFA:SFA ratio; 8.↓ red and processed meat; 9.↔ alcohol;	Age, total energy intake, BMI, smoking status, physical activity, education, age at menarche, age at first live birth, parity, age at menopause, family history of breast cancer, estrogen and progestin use	Breast cancer HR 1.01 (0.94, 1.09) for fifth versus first quintile
Cheng et al. (2018)	USA IWHS	Prospective cohort	Cancer mortality	35,221 17.1 years	55–69	W	aMED score range: 9–45. 1.↑ vegetables; 2.↑ fruits; 3.↑ nuts; 4.↑ legumes; 5.↑ fish; 6.↑ whole grains; 7.↑ MUFA:SFA ratio; 8.↓ red and processed meat; 9.↔ alcohol;	Age, smoking status, education, BMI, physical activity, total energy intake, hormone replacement therapy use, marital status, chronic disease	Cancer mortality HR 0.93 (0.84, 1.03) for fifth versus first quintile
Cheng et al. (2018)	USA IWHS	Prospective cohort	Colorectal cancer	35,221 17.1 years	55–69	W	MDS score range: 11–15. 1.↑ vegetables; 2.↑ fruits; 3.↑ lean meats; 4.↑ fish; 5.↑ nuts; 6.↑ MUFA:SFA ratio; 7.↓ red and processed meat; 8.↓ sodium; 9.↔ dairy foods; 10.↔ grains and starches; 11.↔ alcohol;	Age, family history of colorectal cancer in a first-degree relative, smoking status, education, BMI, physical activity, total energy intake, arthritis, hormone replacement therapy use	Colorectal cancer HR 1.01 (0.86, 1.18) for fifth versus first quintile
Gardeazabal et al. (2020)	Spain SUN	Prospective cohort	Breast cancer	10,713 10.3 years	18–101	W	PCA-derived score. ↑ Vegetables; ↑ fruits ↑ legumes; ↑ nuts; ↑ eggs; ↑ fish; ↑ natural fruit juices; ↑ processed meats; ↑ unprocessed red meat, ↑ poultry; ↑ olive oil; ↑ olive oil; ↑ other fruits;	Age, height, smoking habit, leisure-time physical activity, alcohol intake, BMI, age of menarche, pregnancies of at least 6 months, pregnancies before the age of 30 years, lifetime breastfeeding, use of hormone replacement therapy, time of use of hormone replacement therapy, years of university studies, family history breast cancer, age at menopause, total energy intake, diabetes, propensity scores	Breast cancer HR 0.64 (0.30, 1.37) for forth versus first quartile
Haridass et al. (2018)	USA CTS	Prospective cohort	Breast cancer	96,959 14.0 years	22–104	W	aMED score range: 0–8. 1.↑ vegetables; 2.↑ fruits; 3.↑ nuts and legumes; 4.↑ fish; 5.↑ whole grains; 6.↑ MUFA:SFA ratio; 7.↓ red and processed meat; 9.↔ alcohol;	Age, race, family history of breast cancer, age at menarche, oral contraceptive use, parity status, smoking status, SES, physical activity, total energy intake, BMI	Breast cancer HR 0.97 (0.89, 1.06) for fifth versus first quintile
Hashemian et al. (2019)	Iran GCS	Prospective cohort	Cancer mortality	42,373 10.6 years	40–75	M/W	aMED score range: 0–9. 1.↑ vegetables; 2.↑ fruits; 3.↑ nuts; 4.↑ legumes; 5.↑ fish; 6.↑ whole grains; 7.↑ MUFA:SFA ratio; 8.↓ red and processed meat; 9.↔ alcohol;	Age, sex, BMI, formal education, place of residence, smoking status, opium use, physical activity, wealth score, marital status, history of hypertension, total energy intake	Cancer mortality HR 0.63 (0.46, 0.85) for fifth versus first quintile
Hoon Lee et al. (2019)	USA NHS HPFS	Prospective cohort	Multiple myeloma	116,983 23.9 years	30–75	M/W	aMED score range: 0–9. 1.↑ vegetables; 2.↑ fruits; 3.↑ nuts; 4.↑ legumes; 5.↑ fish; 6.↑ whole grains; 7.↑ MUFA:SFA ratio; 8.↓ red and processed meat; 9.↔ alcohol;	Age, total energy intake, BMI	Multiple myeloma HR 0.97 (0.88, 1.07) for upper versus lower tertile
Hoon Lee et al. (2020)	USA NHS HPFS	Prospective cohort	All-cause mortality among multiple myeloma survivors	423 3.5 years	30–75	M/W	aMED score range: 0–9. 1.↑ vegetables; 2.↑ fruits; 3.↑ nuts; 4.↑ legumes; 5.↑ fish; 6.↑ whole grains; 7.↑ MUFA:SFA ratio; 8.↓ red and processed meat; 9.↔ alcohol;	Age at diagnosis, pre-diagnosis energy intake, pre-diagnosis BMI, time between food frequency questionnaire return date and multiple myeloma diagnosis, year of diagnosis, comorbidity score	All-cause mortality among multiple myeloma survivors HR 0.60 (0.44, 0.83) for upper versus lower tertile
Karavasiloglou et al. (2019)	USA NHANES III	Prospective cohort	All-cause mortality among cancer survivors	230 16.0 years	44.0 (mean)	W	MDS score range: 0–9. 1.↑ vegetables; 2.↑ fruits and nuts; 3.↑ cereals; 4.↑ legumes; 5.↑ fish and seafood; 6.↑ MUFA:SFA ratio; 7.↓ dairy products; 8.↓ meat and processed meat; 9.↔ alcohol;	Age at questionnaire completion, race/ethnicity, time from completion to diagnosis, BMI, marital status, socioeconomic status, smoking status, self-reported prevalent disease at baseline, daily energy consumption, moderate to vigorous activity	All-cause mortality among cancer survivors HR 0.67 (0.41, 1.11) for aMED ≥ 5 versus ≤ 4
Lavalette et al. (2018)	France NNSS	Prospective cohort	Breast cancer Prostate cancer Colorectal cancer	41,543 3.0 years	≥ 40	M/W	MEDI-LITE score range: 0–18. 1.↑ fruits; 2.↑ vegetables; 3.↑ legumes; 4.↑ cereals; 5.↑ fish; 6.↑ olive oil; 7.↓ meat and meat products; 8.↓ dairy products; 9.↔ alcohol;	Age, sex, educational level, smoking status, number of 24-h dietary recalls, height, family history of cancer, BMI, physical activity (further for breast cancer: number of biological children, menopausal status, hormonal treatment for menopause, oral contraception use at baseline)	Breast cancer HR 1.13 (0.84, 1.53) Prostate cancer HR 0.95 (0.61, 1.50) Colorectal cancer HR 1.02 (0.51, 2.04) for fifth versus first quintile
Ma et al. (2019)	USA NHS HPFS	Prospective cohort	Hepatocellular cancer	138,688 < 32.0 years	30–75	M/W	aMED score range: 0–9. 1.↑ vegetables; 2.↑ fruits; 3.↑ nuts; 4.↑ legumes; 5.↑ fish; 6.↑ whole grains; 7.↑ MUFA:SFA ratio; 8.↓ red and processed meat; 9.↔ alcohol;	Age, race, cohort, physical activity level, BMI, smoking, regular aspirin use, total calorie intake, type 2 diabetes	Hepatocellular cancer HR 0.75 (0.49, 1.15) for upper versus lower tertile
Mahamat-Saleh et al. (2019)	France E3N	Prospective cohort	Skin cancer	67,322 15.0 years	40–65	W	MD score range: 0–9. 1.↑ fruits; 2.↑ vegetables; 3.↑ legumes; 4.↑ cereal products; 5.↑ fish; 6.↑ olive oil; 7.↓ meat products; 8.↓ dairy; 9.↔ alcohol;	Age, birth cohort, skin sensitivity to sun exposure, number of nevi, number of freckles, skin color, hair color, family history of skin cancer, level of residential sun exposure at birth and at baseline, energy intake, BMI, physical activity, smoking status, education level, coffee intake	Skin cancer HR 0.83 (0.73, 0.93) for MD ≥ 6 versus ≤ 3
Neelakantan et al. (2018)	China SCHS	Prospective cohort	Cancer mortality	57,078 17.0 years	45–74	M/W	aMED score range: 0–9. 1.↑ vegetables; 2.↑ fruits; 3.↑ nuts; 4.↑ legumes; 5.↑ fish; 6.↑ whole grains; 7.↑ MUFA:SFA ratio; 8.↓ red and processed meat; 9.↔ alcohol;	Age, sex, total energy intake, dialect, level of education, smoking status, sleep duration, BMI, history of diabetes mellitus, history of hypertension	Cancer mortality HR 0.88 (0.80, 0.97) for fifth versus first quintile
Petimar et al. (2019)	USA SS	Prospective cohort	Breast cancer	45,626 7.6 years	35–74	W	aMED score range: 0–9. 1.↑ vegetables; 2.↑ fruits; 3.↑ nuts; 4.↑ legumes; 5.↑ fish; 6.↑ whole grains; 7.↑ MUFA:SFA ratio; 8.↓ red and processed meat; 9.↔ alcohol;	Total energy intake, race/ethnicity, income, smoking, BMI, physical activity, height, education, alcohol intake, mother diagnosed with breast cancer, age at first live birth, parity, hormone replacement therapy, age at menopause, oral contraception use, age at menarche, lifetime duration of breastfeeding, time of last mammogram	Breast cancer HR 0.89 (0.76, 1.05) for forth versus first quartile
Petimar et al. (2018)	USA NHS HPFS	Prospective cohort	Colorectal cancer	124,707 22.3 years	30–75	M/W	aMED score range: 0–9. 1.↑ vegetables; 2.↑ fruits; 3.↑ nuts; 4.↑ legumes; 5.↑ fish; 6.↑ whole grains; 7.↑ MUFA:SFA ratio; 8.↓ red and processed meat; 9.↔ alcohol;	Total energy intake, alcohol intake, physical activity, NSAID use, family history of CRC, previous CRC screening via colonoscopy or sigmoidoscopy, history of polyps, smoking, multivitamin use, supplemental calcium intake, young adult BMI (further for ♂ menopausal status, postmenopausal hormone use)	Colorectal cancer HR 0.91 (0.79, 1.05) for fifth versus first quintile
Ratjen et al. (2017)	Germany PopGen	Prospective cohort	All-cause mortality among colorectal cancer survivors	1404 7.0 years	55–66 (IQR age)	M/W	MMDS score range: 0–9. 1.↑ vegetables; 2.↑ fruits and nuts; 3.↑ legumes; 4.↑ cereals; 5.↑ fish; 6.↑ MUFA + PUFA:SFA ratio; 7.↓ meat and poultry products; 8.↓ dairy; 9.↔ alcohol;	Age at diet assessment, sex, BMI, physical activity, survival time from CRC diagnosis until diet assessment, tumor location, occurrence of metastases, occurrence of other cancer, chemotherapy, smoking status, total energy intake, interactions of time with age, BMI, metastases	All-cause mortality among colorectal cancer survivors HR 0.48 (0.32, 0.74) for fourth versus first quartile
Schulpen et al. (2019)	The Netherlands NLCS	Case-cohort	Colorectal cancer Esophageal cancer (by subtypes) Gastric cancer (by subtypes) Pancreatic cancer Lung cancer Prostate cancer Bladder cancer	120,852 20.3 years	55–69	M/W	aMED score range: 0–9. 1.↑ vegetables; 2.↑ fruits; 3.↑ nuts; 4.↑ legumes; 5.↑ fish; 6.↑ whole grains; 7.↑ MUFA:SFA ratio; 8.↓ red and processed meat; 9.↔ alcohol; aMEDr score range 0–8 Components of aMED excluding alcohol intake mMED score range 0–9. 1.↑ vegetables; 2.↑ fruits and nuts; 3.↑ legumes; 4.↑ cereals; 5.↑ fish; 6.↑ MUFA + PUFA:SFA ratio; 7.↓ meat and poultry products; 8.↓ dairy; 9.↔ alcohol; mMEDr score range 0–8. Components of mMED excluding alcohol intake	Age at baseline, cigarette smoking status, cigarette smoking frequency, cigarette smoking duration, BMI, daily energy intake, highest level of education, non-occupational physical activity, (family history of colorectal, esophageal, gastric, pancreatic, lung, prostate, bladder cancer, respectively)	Colorectal cancer aMED HR 1.03 (0.89, 1.19) Esophageal cancer HR 0.99 (0.64, 1.53) Gastric cancer HR 0.60 (0.44, 0.82) Pancreatic cancer HR 0.89 (0.63, 1.26) Lung cancer HR 0.86 (0.70, 1.05) Prostate cancer HR 1.19 (1.02, 1.40) Bladder cancer HR 0.99 (0.83, 1.18) for aMED ≥ 6 versus ≤ 3
Seon Kuan et al. (2019)	UK MWS USA NIH-AARP PLCO	Prospective cohorts	Glioma	1,262,104 12.2 years	50–74	M/W	aMED score range: 0–9. 1.↑ vegetables; 2.↑ fruits; 3.↑ nuts; 4.↑ legumes; 5.↑ fish; 6.↑ whole grains; 7.↑ MUFA:SFA ratio; 8.↓ red and processed meat; 9.↔ alcohol;	Height, BMI, smoking, alcohol intake, level of educational attainment, region of residence, parity, oral contraceptive use, use of menopausal hormones (for women)	Glioma RR 1.24 (1.05, 1.45) for aMED ≥ 7 versus ≤ 2
Sharma et al. (2018)	Canada NFCC	Prospective cohort	All-cause mortality among colorectal cancer survivors	532 6.3 years	20–75	M/W	aMED score range: 0–9. 1.↑ vegetables; 2.↑ fruits; 3.↑ nuts; 4.↑ legumes; 5.↑ fish; 6.↑ whole grains; 7.↑ MUFA:SFA ratio; 8.↓ red and processed meat; 9.↔ alcohol;	Energy, stage of cancer, sex, age, marital status, tumor location, screening history, intake of alcohol, radiation and chemotherapy status	All-cause mortality among colorectal cancer survivors HR 0.62 (0.39, 0.96) for fourth versus first quartile
Solans et al. (2019)	Europe (10 countries) 10 countries	Prospective cohort	Lymphoma (by molecular subtypes)	476,160 13.9 years	30–70	M/W	arMED score range: 0–16. 1.↑ fruits, nuts and seeds; 2.↑ vegetables; 3.↑ legumes; 4.↑ fish and seafood; 5.↑ olive oil; 6.↑ cereals; 7.↓ dairy products; 8.↓ meat; aMED score range 0–18. Components of arMED and alcohol	Age, center, sex, BMI, total energy intake, educational level, height, physical activity, smoking status, alcohol intake (for arMED)	Lymphoma arMED HR: 0.91 (0.80, 1.03) for high (arMED ≥ 10) versus low (≤ 5) adherence
Sotos-Prieto et al. (2017)	USA NHS HPFS	Prospective cohort	Cancer mortality	73,739 12.0 years	30–75	M/W	aMED score range: 0–9. 1.↑ vegetables; 2.↑ fruits; 3.↑ nuts; 4.↑ legumes; 5.↑ fish; 6.↑ whole grains; 7.↑ MUFA:SFA ratio; 8.↓ red and processed meat; 9.↔ alcohol;	Age, initial dietary score, race, family history of myocardial infarction, diabetes or cancer, use or nonuse of aspirin or multivitamins, initial BMI, initial smoking status and changes in smoking status, initial smoking pack-years and changes in smoking pack-years among participants with any history of smoking, initial levels of physical activity and total energy intake and changes in these levels, menopausal status, use or nonuse of hormone-replacement therapy (for women), history of hypertension, hypercholesterolemia or type 2 diabetes, weight change, use or nonuse of cholesterol-lowering and antihypertensive medications	Cancer mortality HR: 0.98 (0.93, 1.03) For 20-percentile increase in score during 12-year period
Torres Stone et al. (2017)	USA NIH-AARP	Prospective cohort	Colorectal cancer	398,458 10.3 years	50–71	M/W	MDS score range: 0–9. 1.↑ vegetables; 2.↑ fruits; 3.↑ nuts; 4.↑ legumes; 5.↑ fish; 6.↑ whole grains; 7.↑ MUFA:SFA ratio; 8.↓ red and processed meat; 9.↔ alcohol;	Age, gender, race-ethnicity, education, smoking, physical activity, energy intake	Colorectal cancer HR: 0.79 (0.71, 0.89) for fifth versus first quintile
Warensjö Lemming et al. (2018)	Sweden SMC	Prospective cohort	Cancer mortality	33,341 17.0 years	40–74	W	mMED score range: 0–8. 1.↑ fruits and vegetables; 2.↑ legumes and nuts; 3.↑ non-refined and high-fiber grains; 4.↑ fermented dairy products; 5.↑ fish; 6.↑ olive or rapeseed oil for cooking or dressing; 7.↓ red and processed meat; 9.↔ alcohol;	Educational level, living alone, physical activity, smoking habits, Charlson’s weighted comorbidity index, Healthy Nordic Food Index	Cancer mortality HR 0.81 (0.69, 0.94) for mMED ≥ 6 versus ≤ 2
Witlox et al. (2019)	Europe, USA, Australia (12 countries) EPIC VITAL NLCS MCCS	Prospective cohort	Bladder cancer	646,222 10.2 years	≥ 18	M/W	MDS score range: 0–9. 1.↑ vegetables; 2.↑ fruits; 3.↑ nuts; 4.↑ legumes; 5.↑ fish; 6.↑ whole grains; 7.↑ MUFA:SFA ratio; 8.↓ red and processed meat; 9.↔ alcohol;	Total energy intake, smoking status, sex, age	Bladder cancer HR 0.85 (0.77, 0.93) for score ≥ 6 versus ≤ 3

Author (year)	Country	Study design	Outcome	Cases/controls	Age at entry (years)	Sex	Components of score	Adjustment	OR/RR (95% CI) Multivariable adjusted
Bravi et al. (2018)	Italy	Case–control	Bladder cancer	690/665	25–84	M/W	MDS score range: 0–9. 1.↑ vegetables; 2.↑ legumes; 3.↑ fruits and nuts; 4.↑ cereals; 5.↑ fish; 6.↑ MUFA:SFA ratio; 7.↓ meat, 8.↓ dairy products; 9.↔ alcohol;	Sex, age, year of interview, study center, years of schooling, smoking, BMI, non-alcohol energy intake, history of diabetes, history of cystitis, family history of bladder cancer	Bladder cancer OR 0.66 (0.47, 0.93) for score ≥ 6 versus ≤ 3
Castello et al. (2018)	Spain	Case–control	Gastric cancer	295/3040	23–85	M/W	PCA-derived score. ↑ vegetables (leafy, fruiting root, other); ↑ potatoes; ↑ fruits; ↑ legumes; ↑ seafood/shellfish; ↑ fish (white and oily); ↑ olives and vegetable oil; ↓ juices;	Sex, age, education, BMI, family history of gastric cancer, physical activity, smoking status, caloric intake, alcohol intake, province of residence	Gastric cancer OR 0.53 (0.34, 0.82) for forth versus first quartile
Castello et al. (2019)	Spain	Case–control	Colorectal cancer	1629/3509	22–85	M/W	PCA-derived score. ↑ vegetables (leafy, fruiting root, other); ↑ potatoes; ↑ fruits; ↑ legumes; ↑ seafood/shellfish; ↑ fish (white and oily); ↑ olives and vegetable oil; ↓ juices;	Sex, age, education, BMI, family history of colorectal cancer, physical activity, smoking status, caloric intake, alcohol intake, province of residence	Colorectal cancer OR 0.65 (0.53, 0.80) for forth versus first quartile
Jafari Nasab et al. (2019)	Iran	Case–control	Colorectal cancer	129/240	30–79	M/W	MSDPS score range: 0–100. 1. whole grain cereals (8 servings/day); 2. fruits (3 servings/day); 3. vegetables (6 servings/day); 4. dairy products (2 servings/day); 5. fish and other seafood (6 servings/week); 6. poultry (4 servings/week); 7. olives/legumes/nuts (4 servings/week); 8. Potatoes and other starchy roots (3 servings/week); 9. eggs (3 servings/week); 10. sweets (3 servings/week); 11. meat (1 serving/week); 12. olive oil (exclusive use);	Age, comorbidity, cancer family history, common ways of cooking, level of salt intake, physical activity, calcium supplement use	Colorectal cancer OR 0.19 (0.09, 0.38) for upper versus lower tertile
Jalilpiran et al. (2018)	Iran	Case–control	Prostate cancer	60/60	63.7 (mean)	M	PCA-derived score. ↑ fruit/fruit juices; ↑ nonstarchy vegetables; ↑ olive; ↑ nuts; ↑ fish; ↑ low-fat dairy;	Age, BMI, total energy intake, physical activity, smoking, job, education, usage of antihyperlipidemic drugs, antihypertensive drugs, and aspirin	Prostate cancer OR 0.62 (0.22, 1.77) for score ≥ median versus < median
Krusinska et al. (2018)	Poland	Case–control	Breast cancer	190/230	40–80	W	Polish-aMED score range: 0–8. 1.↑ vegetables; 2.↑ fruit; 3.↑ wholemeal cereals; 4.↑ fish; 5.↑ legumes; 6.↑ nuts and seeds; 7.↑ ratio of vegetable oils to animal fat, 8.↓ red and processed meat;	Age, BMI, socioeconomic status, overall physical activity, smoking status, abuse of alcohol, age at menarche, menopausal status, number of children, oral contraceptive use, hormone-replacement therapy, family history of breast cancer in first- or second-degree relative, vitamin/mineral supplements use, molecular of breast cancer subtypes	Breast cancer OR 0.52 (0.25, 1.07) for score ≥ 6 versus ≤ 2
Ricceri et al. (2017)	Italy	Case–control	Endometrial cancer	297/307	40–74	W	MD score range: 0–8. 1.↑ legumes; 2.↑ cereals; 3.↑ fruits; 4.↑ vegetables; 5.↑ MUFA:SFA ratio; 6.↓ meat and meat products; 7.↓ milk and dairy products, 8.↔ alcohol;	Age, parity, menopausal status, hormone replacement therapy use, oral contraceptive use, BMI, age at menarche, physical activity, education, smoking status, total energy intake	Endometrial cancer OR 0.51 (0.28, 0.92) for score ≥ 6 versus ≤ 3
Russo et al. (2019)	Italy	Case–control	Prostate cancer	118/238	68.7 (mean)	M	MEDI-LITE score range: 0–18. 1.↑ fruits; 2.↑ vegetables; 3.↑ legumes; 4.↑ cereals; 5.↑ fish; 6.↑ olive oil; 7.↓ meat and meat products; 8.↓ dairy products; 9.↔ alcohol;	Age, energy intake, weight status, smoking status, alcohol consumption, physical activity level, family history of prostatic cancer, total polyphenol intake	Prostate cancer OR 0.16 (0.03, 0.72) for score > 7 versus ≤ 3
Saraiya et al. (2020)	USA	Case–control	Head and neck cancer	1170/1303	20–80	M/W	MDS score range: 0–9. 1.↑ vegetables; 2.↑ legumes; 3.↑ fruits; 4.↑ cereals/grains; 5.↑ fish; 6.↑ MUFA:SFA ratio; 7.↓ meat, 8.↓ dairy products; 9.↔ alcohol;	Age, race, sex, BMI, history of loose teeth, educational attainment, lifetime number of years smoking cigarettes, quartile of lifetime intake of alcohol, quartile of energy intake	Head and neck cancer OR 0.88 (0.80, 0.98) for 1-SD increase in score
Salvatore Benito et al. (2019)	Italy	Case–control	Head and neck cancer	68/100	61.8 (mean)	M/W	MEDAS screener range: 0–14. 1.↑ olive oil (as primary source of fat); 2.↑ olive oil; 3.↑ vegetables; 4.↑ fruits; 5.↑ wine; 6.↑ legumes; 7.↑ fish/seafood; 8.↑ nuts; 9.↑ chicken, turkey, rabbit (as preferred meat); 10.↑ sofrito dishes; 11.↓ red meat/hamburger/sausages; 12.↓ butter/margarine/cream; 13.↓ carbonated/sugar-sweetened beverages; 14.↓ commercial pastry;	Age, gender, smoking, alcohol income level, education level	Head and neck cancer OR 0.48 (0.20, 1.07) for score ≥ 8 versus < 8
Solans et al. (2018)	Spain	Case–control	Chronic lymphocytic leukemia	369/1605	20–85	M/W	PCA-derived score. ↑ vegetables (leafy, fruiting root, other); ↑ potatoes; ↑ fruits; ↑ legumes; ↑ seafood/shellfish; ↑ fish (white and oily); ↑ olives and vegetable oil; ↓ juices;	Age, sex, education, energy intake, province of residence	Chronic lymphocytic leukemia OR 0.89 (0.61, 1.29) for forth versus first quartile
Turati et al. (2018)	Italy, Switzerland	Case–control	Breast cancer	3034/3392	19–79	W	MDS score range: 0–9. 1.↑ vegetables; 2.↑ legumes; 3.↑ fruits and nuts; 4.↑ cereals; 5.↑ fish; 6.↑ MUFA:SFA ratio; 7.↓ meat, 8.↓ dairy products; 9.↔ alcohol;	Study centre, age, education, BMI, physical activity, smoking, parity, menopausal status, oral contraceptive use, hormone-replacement therapy use, diabetes, family history of breast cancer, non-alcohol energy intake	Breast cancer OR 0.82 (0.71, 0.95) for score ≥ 6 versus ≤ 3

*BMI* Body Mass Index, *CI* confidence interval, *CRC* colorectal cancer, *HR* hazard ratio; *M* men, *NSAID* non-steroid autoinflammatory drugs, *OR* odds Ratiom, *RR* risk ratio, *W* women

↑ High intake; ↓ Low intake; ↔ Moderate intake; *aMDS* adapted Mediterranean diet score, *aMED* alternate Mediterranean diet; *arMED* adapted relative Mediterranean diet, *MD* Mediterranean diet, *MDS* Mediterranean diet score, *MEDAS* Mediterranean Diet Adherence Screener, *MEDI-LITE* Mediterranean diet based on the literature, *MMDS* modified Mediterranean diet score, *mMED* modified Mediterranean diet, *MSDPS* Mediterranean-Style Dietary Pattern Score,

*CTS* California Teachers Study, *E3N* Etude Epidémiologique auprès de femmes de la Mutuelle Générale de l'Education Nationale, *EPIC* European Prospective Investigation into Cancer and Nutrition, *GCS* Golestan Cohort Study, *HPFS* Health's Professional Follow-up Study, *IWHS* Iowa Women's Health Study, *MCCS* Melbourne Collaborative Cohort Study, *MEC* Multiethnic Cohort Study, *MS* Moli-sani Study, *MWS* UK Million Women Study, *NHANES* National Health and Nutrition Examination Survey,  *NFCC* Newfoundland Familial Colorectal Cancer Cohort, *NHS* Nurses' Health Study, *NIH-AARP* National Institute of Health–American Association of Retired Persons Study, *NLCS* Netherlands Cohort Study, *NNSS* NutriNet-Santé Cohort, *PLCO* Prostate, Lung, Colorectal and Ovarian Cancer Screening Trial, *SCHS* Singapore Chinese Heath Study, *SMC* Swedish Mammography Cohort, *SS* Sister Study, *SUN* Seguimiento University of Navarra, *VIP* Västerbotten Intervention Programme, *VITAL* Vitamins and Lifestyle Study

### Definitions of Mediterranean diet

The majority of 44 newly included studies assessed adherence to the MedDiet using predefined dietary scores. Two main definitions of MedDiet used in the included studies referred to scores by Trichopoulou [12] and Fung [26]. Fewer reports adopted scores by Sofi [150] and Buckland [55]. Differences between scores concerned mostly cut-off points for moderate alcohol consumption and a way of handling healthy fat intake. Five added studies derived MedDiet scores from principal component analysis [113, 114, 116, 122, 125].

Corresponding risk estimates were based on comparison of extreme quantiles (top quintile/quartile/tertile versus bottom) [17, 18, 113–116, 122, 124–126, 128–140], fixed cut-off points [20, 21, 112, 117–120, 123, 141–149], per standard deviation [121], per-tertile [127] or per-20 percentile increase in the MedDiet score [19]. Majority of studies used MedDiet scores evaluated in the baseline, whereas one study reported risk in the context of a 12-year change of adherence to the dietary pattern [19].

### Main outcomes

According to the different clinical outcomes, risk of cancer mortality was evaluated in 18 cohort studies and one RCT (*n* = 71,145 cases); breast cancer risk in 12 cohort, one RCT (*n* = 35,373 incident cases) and 11 case–control studies (*n* = 10,615 prevalent cases); colorectal cancer risk in nine cohort, one case–cohort (*n* = 26,185 incident cases) and seven case–control studies (*n* = 9683 prevalent cases); prostate cancer risk in five cohort, one case–cohort (*n* = 36,006 incident cases) and five case–control studies (*n* = 2466 prevalent cases); respiratory cancer risk in four cohort and one case–cohort studies (*n* = 12,730 incident cases); gastric cancer risk in three cohort, one case–cohort (*n* = 2343 incident cases) and three case–control studies (*n* = 1517 prevalent cases); liver cancer risk in three cohort (*n* = 1274 incident cases) and one case–control study (*n* = 518 prevalent cases); bladder in three cohort (*n* = 5844 incident cases) and one case–control study (*n* = 690 prevalent cases); pancreatic cancer risk in two cohort, one case–cohort (*n* = 1436 incident cases) and one case–control study (*n* = 688 prevalent cases); blood cancer risk in two cohort (*n* = 3614 incident cases) and two case–control studies (*n* = 691 prevalent cases); esophageal cancer in one cohort, one case–cohort (*n* = 1181 incident cases) and one case–control study (*n* = 304 prevalent cases); head and neck in one cohort (*n* = 1868 incident cases) and eight case–control studies (*n* = 4601 prevalent cases); endometrial cancer in one cohort (*n* = 1392 incident cases) and three case–control studies (*n* = 2355 prevalent cases); biliary tract (*n* = 163 incident cases), gallbladder (*n* = 77 incident cases), ovarian (*n* = 696 incident cases), skin cancer (*n* = 1436 incident cases) and glioma risk (*n* = 2313 incident cases) in one cohort study, respectively. Among cancer survivors, eight cohort studies summarized all-cause mortality (*n* = 4883 cases), cancer-specific mortality in four cohort studies (*n* = 1790 cases), and cancer reoccurrence in one cohort study (*n* = 92 cases).

Pooled estimates from random-effects models are summarized in Table 2 and corresponding forest plots are presented in ESM Figs. 2–22. Highest versus lowest adherence to the MedDiet was associated with a lower risk of cancer mortality in cohort studies (RR_cohort_: 0.87, 95% CI 0.82–0.92; *I*^2^ = 83%), but not in one RCT (RR_RCT_: 0.75, 95% CI 0.17–3.33, *I*^2^ = NA). Among cancer survivors, there was no association between the adherence to the MedDiet and cancer mortality risk (RR_cohort_: 0.96, 95% CI 0.82–1.11; *I*^2^ = 0%); however, an inverse association was observed in relation to all-cause mortality (RR_cohort_: 0.75, 95% CI 0.66–0.86, *I*^2^ = 41%). An inverse association of breast cancer with highest adherence to the MedDiet was found in one RCT (RR_RCT_: 0.41, 95% CI 0.19–0.87, *I*^2^ = NA) and observational studies (RR_observational_: 0.94, 95% CI 0.90–0.97, *I*^2^ = 31%). However, considering separate designs there was a risk reduction in case–control studies (OR_case–control_: 0.87, 95% CI 0.82–0.93, *I*^2^ = 6%), but not in cohort studies (RR_cohort_: 0.97, 95% CI 0.94–1.00, *I*^2^ = 0%). Regarding colorectal cancer, the highest adherence to the MedDiet was linked to a reduced risk (RR_observational_: 0.83, 95% CI 0.76–0.90, *I*^2^ = 82%; OR_case–control_: 0.64, 95% CI 0.52–0.79, *I*^2^ = 89%, RR_cohort_: 0.92, 95% CI 0.87–0.99, *I*^2^ = 50%). Furthermore, inverse associations between adherence to the MedDiet and risk of head and neck (RR_observational_: 0.56, 95% CI 0.44–0.72; *I*^2^ = 91%, OR_case–control_: 0.54, 95% CI 0.40–0.72, *I*^2^ = 92%; RR_cohort_: 0.73, 95% CI 0.60–0.89, *I*^2^ = NA), bladder (RR_observational_: 0.87, 95% CI 0.76–0.98; *I*^2^ = 38%, OR_case–control_: 0.66, 95% CI 0.47–0.93, *I*^2^ = NA; RR_cohort_: 0.89, 95% CI 0.81–0.97, *I*^2^ = 11%), gastric (RR_observational_: 0.70, 95% CI 0.61–0.80; *I*^2^ = 52%, OR_case–control_: 0.63 95% CI 0.53–0.75, *I*^2^ = 34%; RR_cohort_: 0.77, 95% CI 0.64–0.92, *I*^2^ = 44%), liver (RR_observational_: 0.64, 95% CI 0.54–0.75, *I*^2^ = 0%; OR_case–control_: 0.51, 95% CI 0.34–0.77, *I*^2^ = NA; RR_cohort_: 0.67, 95% CI 0.56–0.80, *I*^2^ = 0%), and respiratory (RR_cohort_: 0.84, 95% CI 0.76–0.94; *I*^2^ = 42%) cancers were found, respectively. Consistently no effect of adhering to the MedDiet was observed in relation to blood (RR_observational_: 0.94, 95% CI 0.88–1.02, *I*^2^ = 0%; OR_case–control_: 0.89, 95% CI 0.68–1.18, *I*^2^ = 0%; RR_cohort_ 0.95, 95% CI 0.88–1.02, *I*^2^ = 0%) and prostate cancer (RR_observational_: 0.98, 95% CI 0.93–1.04, *I*^2^ = 39%; OR_case–control_: 0.76, 95% CI 0.52–1.13, *I*^2^ = 6%; RR_cohort_ 0.98, 95% CI 0.94–1.02, *I*^2^ = 28%). Additionally no associations were observed for endometrial, esophageal and pancreatic cancer in observational studies (RR_observational_: 0.67, 95% CI 0.41–1.11, *I*^2^ = 91%; RR_observational_: 0.64, 95% CI 0.35–1.16, *I*^2^ = 81%; RR_observational_: 0.80, 95% CI 0.60–1.06, *I*^2^ = 79%, respectively) with contrary findings from cohort (RR_cohort_: 0.98, 95% CI 0.82–1.17, *I*^2^ = NA; RR_cohort_: 0.85, 95% CI 0.67–1.09, *I*^2^ = 0%; RR_cohort_: 0.92, 95% CI 0.81–1.05, *I*^2^ = 0%, respectively) and case–control studies (OR_case–control_: 0.58, 95% CI 0.35–0.95, *I*^2^ = 77%; OR_case–control_: 0.26, 95% CI 0.13–0.52, *I*^2^ = NA; OR_case–control_: 0.48, 95% CI 0.35–0.66, *I*^2^ = NA, respectively).

**Table 2:** Pooled relative risk of cancer mortality, site-specific cancers and outcomes among cancer survivors for highest versus lowest adherence to Mediterranean dietary pattern

Outcomes	Case–control studies				Cohort studies				Observational studies				Randomized controlled trials				NutriGrade assessment	
	*N*	OR	95% CI	*I*^2^ (%)	*N*	RR	95% CI	*I*^2^ (%)	*N*	RR	95% CI	*I*^2^ (%)	*N*	RR	95% CI	*I*^2^ (%)	Cohort studies	RCTs
Cancer mortality	–	–	–	–	18	0.87	0.82, 0.92	83	–	–	–	–	1	0.75	0.17, 3.33	NA	Moderate	Low
Biliary tract cancer	–	–	–	–	1	0.44	0.29, 0.67	NA	–	–	–	–	–	–	–	–	Very low	–
Bladder cancer	1	0.66	0.47, 0.93	NA	3	0.89	0.81, 0.97	11	4	0.87	0.76, 0.98	38	–	–	–	–	Low	–
Blood cancer	2	0.89	0.68, 1.18	0	2	0.95	0.88, 1.02	0	4	0.94	0.88, 1.02	0	–	–	–	–	Low	–
Breast cancer	11	0.87	0.82, 0.93	6	12	0.97	0.94, 1.00	0	23	0.94	0.90, 0.97	31	1	0.41	0.19, 0.87	NA	Low	Low
Colorectal cancer	7	0.64	0.52, 0.79	89	10	0.92	0.87, 0.99	50	17	0.83	0.76, 0.90	82	–	–	–	–	Moderate	–
Endometrial cancer	3	0.58	0.35, 0.95	77	1	0.98	0.82, 1.17	NA	4	0.67	0.41, 1.11	91	–	–	–	–	Very low	–
Esophageal cancer	1	0.26	0.13, 0.52	NA	2	0.85	0.67, 1.09	0	3	0.64	0.35, 1.16	81	–	–	–	–	Very low	–
Gallbladder cancer	–	–	–	–	1	0.42	0.23, 0.77	NA	–	–	–	–	–	–	–	–	Very low	–
Gastric cancer	3	0.63	0.53, 0.75	34	4	0.77	0.64, 0.92	44	7	0.70	0.61, 0.80	52	–	–	–	–	Low	–
Glioma	–	–	–	–	1	1.24	1.05, 1.45	NA	–	–	–	–	–	–	–	–	Very low	–
Head and neck cancer	8	0.54	0.40, 0.72	92	1	0.73	0.60, 0.89	NA	9	0.56	0.44, 0.72	91	–	–	–	–	Very low	–
Liver cancer	1	0.51	0.34, 0.77	NA	3	0.67	0.56, 0.80	0	4	0.64	0.54, 0.75	0	–	–	–	–	Low	–
Ovarian cancer	–	–	–	–	1	0.91	0.71, 1.17	NA	–	–	–	–	–	–	–	–	Very low	–
Pancreatic cancer	1	0.48	0.35, 0.66	NA	3	0.92	0.81, 1.05	0	4	0.80	0.60, 1.06	79	–	–	–	–	Very low	–
Prostate cancer	5	0.76	0.52, 1.13	56	6	0.98	0.94, 1.02	28	11	0.98	0.93, 1.04	39	–	–	–	–	Low	–
Respiratory cancer	–	–	–	–	5	0.84	0.76, 0.94	42	–	–	–	–	–	–	–	–	Low	–
Skin cancer	–	–	–	–	1	0.83	0.73, 0.93	NA	–	–	–	–	–	–	–	–	Very low	–
All–cause mortality among survivors	–	–	–	–	8	0.75	0.66, 0.86	41	–	–	–	–	–	–	–	–	Low	–
Cancer mortality among survivors	–	–	–	–	4	0.96	0.82, 1.11	0	–	–	–	–	–	–	–	–	Very low	–
Cancer reoccurrence among survivors	–	–	–	–	1	0.61	0.18, 2.07	NA	–	–	–	–	–	–	–	–	Very low	–

*CI* confidence interval, *I*^2^ percentage of variation across studies due to heterogeneity, *N* number of studies, *RCTs* randomized controlled trials

### Subgroup analysis

None of the effect estimates was modified by the type of MedDiet score or geographical localization of study. Both menopausal status neither receptor expression pattern did not change the effect estimate for breast cancer. By specifying anatomical location of colorectal cancer, the general inverse association was re-established for distal colon and rectum (RR: 0.88, 95% CI 0.79 to 0.96, *I*^2^ = 0% and RR: 0.86, 95% CI 0.75–0.98, *I*^2^ = 42%, respectively), but not for proximal colon (RR: 1.01, 95% CI 0.93–1.09, *I*^2^ = 0%). The corresponding effect estimates are summarized in ESM Tables 2–4.

### Components of the MedDiet and risk of cancer

**Fig. 1:**
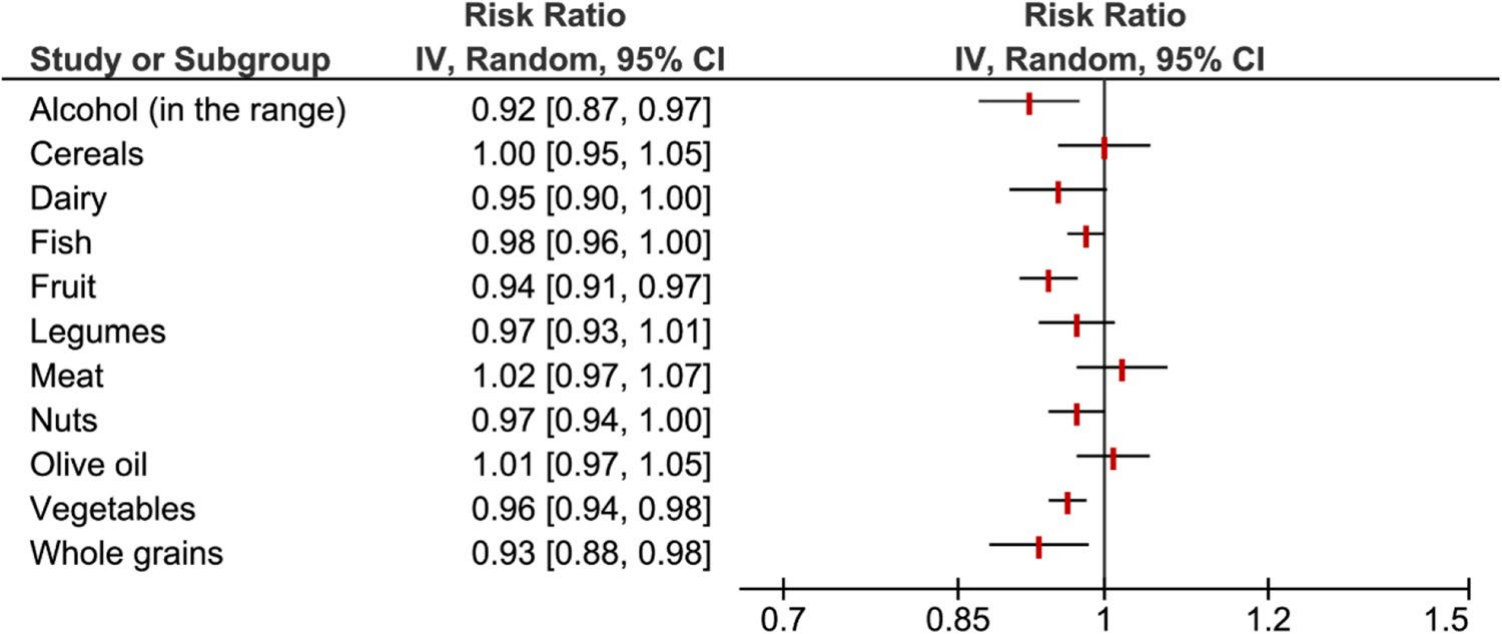
Pooled risk ratios of individual Mediterranean diet components and overall cancer risk

Summary risk ratios for the components of the MedDiet score are presented in Fig. 1. We found an inverse association between alcohol consumption within the recommended range compared to higher consumption (RR: 0.92, 95% CI 0.87–0.97), whole grain intake (RR: 0.93, 95% CI 0.88–0.98), fruit intake (RR: 0.94, 95% CI 0.91–0.97) as well as vegetable intake (RR: 0.96, 95% CI 0.94–0.98) and overall cancer risk. No associations were identified for cereals, dairy, fish, legumes, meat, nuts, and olive oil.

### Publication bias

The results of Egger’s linear regression test did not support the presence of publication bias for cancer mortality (*P* = 0.55), breast cancer (*P* = 0.94), and colorectal cancer (*P* = 0.74) following comparison between highest and lowest adherence to the MedDiet. Funnel plots were created for analyses including at least 10 studies. Visual inspection of the plots suggested low asymmetry for colorectal cancer, as well as moderate asymmetry for cancer mortality and breast cancer, implying that publication bias might be affecting these associations (ESM Figs. 23–25).

### Certainty of evidence

Application of the NutriGrade tool to the results from cohort studies resulted in moderate certainty of evidence for cancer mortality and colorectal cancer risk. Low certainty of evidence was found for incidence of bladder, blood, breast, gastric, liver, prostate and respiratory cancer as well as all-cause mortality among survivors. In RCTs certainty of evidence for breast cancer and cancer mortality was low. The credibility of findings for remaining site-specific cancers, cancer mortality and reoccurrence among cancer survivors was rated as very low, suggesting very low confidence in effect estimates (Table 2, ESM Table 5).

## Discussion

In this updated systematic review, we meta-analysed current evidence on the association between adherence to MedDiet pattern and the risk of cancer. We identified 44 new studies, which provided data for an additional one million participants. The present analysis confirmed our previous findings on the inverse association of adherence to MedDiet on cancer mortality and colorectal cancer risk [16]. Contrary to our earlier reports, we observed conflicting findings between case–control and cohort studies for breast cancer [16]. Lack of association in cohort studies might suggest that significant findings found in case–control studies could be explained by bias linked to study design. Therefore, we cannot conclude on presence of inverse association between MedDiet and breast cancer risk. Our finding corresponds with statement from Continuous Updated Project by the WCRF suggesting too limited evidence to draw a conclusion on the relationship of healthy dietary patterns and breast cancer [10]. For the first time, we were able to observe an inverse association between adherence to the MedDiet and bladder, gastric and lung cancer incidence, as well as all-cause mortality among cancer survivors. Moreover, the present report included new cancer subtypes such as skin cancer and glioma, as well as identified new studies for those comparisons represented previously by a single study. The certainty of the evidence, evaluated for the first time in these series of reviews, was judged as “moderate” for cancer mortality and colorectal cancer and “very low” to “low” for other cancer subtypes. The NutriGrade scoring system did consider only meta-analyses of RCTs and cohort studies [23], but not case–control studies. Similarly, the evidence which was the basis for the 3rd WCRF report, considered only RCTs, cohort studies, and nested case–control studies. Individual case–control studies were not anymore considered due to limitation such as recall bias [10]. Considering the fact that in the current update we were able to identify only two RCTs with a very limited sample size, a major focus when interpreting our results should be put on findings from cohorts with a supportive role of case–control studies.

In 2014, Fardet and Rock referred to analyses of single nutrients or food groups as a reductionist approach not adequate in studies on the preventive effects of nutrition in chronic diseases such as cancer [151]. In contrast, dietary patterns may take into account synergistic and antagonistic interactions between the components of a food matrix, thus yielding a holistic net effect of diet [9]. Adherence to high-quality diets, such as the Healthy Eating Index (HEI) or the alternate HEI was inversely associated with cancer risk by approximately 15% [152]. In our analyses, the beneficial associations of the complete MedDiet on cancer was only reflected by inverse associations between overall cancer risk and fruit, vegetables, whole grains, and moderate alcohol intake, but not for fish, dairy and nuts (Fig. 1). Nevertheless, these observations may provide some insights to explain the mechanisms of action of MedDiet components/bioactive substances [153].

Fruits, vegetables, legumes and whole-grain products are a rich source of dietary fibre. Strong evidence from observational studies suggests a protective role of fibre mostly against colorectal cancer [10]. However, higher intake of dietary fibre was linked to a reduced risk of several other types of neoplasms including breast, gastric and lung cancer as well [154–156]. Gut microbiota reduces digested fibre to short-chain fatty acids such as butyrate, which helps to maintain proper function of the intestinal epithelium, as well as to reduce oncogenic potential by inducing cell apoptosis [157]. Some experimental studies demonstrated a direct interaction between fibre and pattern recognition receptors modulating immune anti-tumour response [158]. By increasing stool bulk, fibre can also dilute and slow absorption of potential carcinogens [158]. In addition, fruits and vegetables provide a variety of phytochemicals with potential anti-cancer effects. Bioactive substances such as carotenoids, flavonoids, stilbenes, coumarins and tannins can act synergically to increase antioxidative capacity and reduce cell oxidative damage [159, 160]. Furthermore, these compounds were shown to inhibit signal-transducing pathways, cell proliferation and oncogene expression, as well as to induce cell-cycle arrest [161]. A meta-analysis of prospective observational studies yielded an inverse association between antioxidative phytochemicals intake or their plasma/serum levels and risk of cancer [162]. Whole grains contain alk(en)ylresorcinols, benzoxanizoids and phytosteroids, which exerted an inhibitory effect on model human cancer cells [163]. Frequent consumption of whole grains was observed to lower risk of cancer mortality and incidence [164–166].

Apart from providing protective compounds, adherence to the MedDiet pattern decreases exposure to potential carcinogens by omitting intake of detrimental food items. Thus, extensive consumption of red and processed meat are associated with an increased risk of cancer, especially colorectal [165, 167]. Both food groups are a potential source of *N*-nitroso compounds, polycyclic aromatic hydrocarbons, and heterocyclic amines known to be cancerogenic [168–170]. A recent meta-analysis suggested that the above-mentioned chemicals are associated both with increased risks of colorectal and gastric cancers [171, 172].

Alcohol predominately in the form of red wine represents the most controversial ´food group´ within the context of the associations of the MedDiet on cancer. Increased ROS synthesis, suppressed anti-tumour immune response as well as metabolization of ethanol into DNA-damaging acetaldehyde may all explain the positive association between alcohol intake and cancer [125, 126]. The 3rd WCRF report indicated that there is a “convincing” grade of evidence for a positive association between alcohol intake and risk of upper aerodigestive, breast, colorectal or liver cancers, irrespective of the type of drink [10]. Definitions of moderate alcohol intake differ between the various MedDiet scores. According to Trichopoulou et al., consumption of up to 50 g/days for men and 25 g/days for women in form of red wine is considered as moderate [12], whereas Fung et al. [92] set cut-off points at 25 g/days and 15 g/days, respectively. Potential anti-tumorigenic effects of red wine are attributed to its polyphenolic content, especially resveratrol [173]. Although our results suggested a small reduction in overall cancer risk for alcohol intake within the range, compared to higher alcohol consumption, the benefit from light-to-moderate consumption of wine on cancer risk in observational studies is inconclusive [174–176]. Risk estimates for several cancers based on MedDiet scores including alcohol did not differ from those simply adjusted for total alcohol intake [122, 145–149]. Consumption of wine together with meals is a part of the cultural heritage in Mediterranean countries, but it is less common in other countries [177]. Therefore, the promotion of wine drinking in countries, where it is not a habit seems pointless, as small benefits do not exclude potential harm.

As already stated in the previous versions of this systematic review, a major limitation of our findings is the inconsistency of the definition of the MedDiet pattern [16]. Initially, the phrase was coined on the basis of observation made in several communities in the Mediterranean basin in the 1960s. Dietary intake has changed significantly since that time, which was stated in follow-up reports from the Seven Countries Study [178]. Therefore, MedDiet should rather be considered as a set of local variants based on cultural setting, food price and availability [177]. Consequently, dietary indices adopted in nutritional epidemiology as a means to quantify adherence to the MedDiet show substantial differences both in the composition of score as well as cut-off points for specific components. A recent umbrella review identified 74 different MedDiet scores used among studies eligible for systematic reviews and meta-analyses [13]. Popular definitions such as traditional MedDiet or alternate MedDiet indices use cut-off points based on median intake in studied populations [12, 26], which may substantially differ between studied populations. For example, median intake of vegetables for men in the Italian subcohort of the EPIC-InterAct study was 291 g/days, whereas the respective value in the Swedish subcohort was only 123 g/days [179]. A potential tool to address dissimilarities between MedDiet scores is the adoption of country-specific food environments [177]. For instance, olive oil, especially EVOO is rarely consumed in the US and northern Europe; therefore, MUFA-to-SFA ratio represents a more suitable measure of healthy fat intake [177]. However, little is known whether the use of these correction factors may result in equivalent preventive effects of MedDiet against cancer. Future studies need to focus on the use of literature-cased cut-off points for food groups as well as on the question whether different adaptations of MedDiet will yield comparable health-related outcomes.

Another limitation is the fact that pooled estimates presented in this review are based predominately on cohort studies set in Europe and the US, whereas single reports covered data from Asia. Uneven distribution of geographical locations might contribute to increased heterogeneity of data due to differences in cancer prevalence, genetic factors, or the burden of environmental risks, which can modify the effect of diet. However, stratifying analyses for study location did not affect the identified risk estimates in the present study.

Our results are based on observational rather than experimental data, which limits the interpretation of our findings with respect to causality. The use of randomized controlled trials in nutrition is limited by the inability to maintain high compliance during the long term of follow-up. Therefore, the use of data from prospective cohort studies is reasonable. To increase trust in our estimates, we did not consider case–control studies, which are prone to recall bias.

Most of the included studies constructed risk models using scores derived from food frequency data assessed during recruitment to study. Diet quality can substantially change during a long follow-up period. Thus, baseline adherence to the dietary pattern does not have to represent the true exposure. A particular strength of our systematic review is the large number of included studies as well as corresponding cancer cases. Another advantage of our analysis was the use of the NutriGrade tool. While assessing the certainty of evidence is key to construct evidence-based recommendations, it is rarely adopted in meta-analyses in nutrition research. To our best knowledge, this systematic review represents the first summary of trustworthiness of associations between adherence to the MedDiet and risk of cancer.

## Conclusion

In conclusion, this systematic review and meta-analysis provides an updated body of evidence on the association between adherence to the MedDiet and risk of cancer. Our results suggest that highest adherence to the MedDiet was inversely associated with risk of cancer mortality in the general population, and all-cause mortality among cancer survivors as well as colorectal, head and neck, respiratory, gastric, liver and bladder cancer risks. However, the very low to moderate certainty of evidence found in this update requires a conservative interpretation of our findings.

## Electronic supplementary material

Below is the link to the electronic supplementary material.

Supplementary file1 (PDF 3563 kb)
