# Supplementary material for: An updated systematic review and meta-analysis on adherence to mediterranean diet and risk of cancer
Source: Eur J Nutr. 2020 Aug 8;60(3):1561–86. doi: 10.1007/s00394-020-02346-6 (PMC7987633; doi:10.1007/s00394-020-02346-6)
Supplement: Supplementary file 1 — Supplementary file1 (PDF 3563 kb) [file 394_2020_2346_MOESM1_ESM.pdf]

Electronic Supplementary Material

Content:

ESM Figure 1. Flowchart of study selection process in the updated systematic review.

ESM Table 1. Reasons for study exclusion at the full-text eligibility assessment step.

ESM Figure 2. Forest plot presenting pooled risk ratio with 95% confidence interval (CI) for all-cause mortality among cancer survivors in cohort studies.

ESM Figure 3. Forest plot presenting pooled risk ratio with 95% confidence interval (CI) for biliary tract cancer in cohort studies.

ESM Figure 4. Forest plot presenting pooled odds and risk ratios with 95% confidence interval (CI) for bladder cancer in case-control and cohort studies.

ESM Figure 5. Forest plot presenting pooled odds and risk ratios with 95% confidence interval (CI) for blood cancer in case-control and cohort studies.

ESM Figure 6. Forest plot presenting pooled odds and risk ratios with 95% confidence interval (CI) for breast cancer in case-control, cohort studies and RCTs.

ESM Figure 7. Forest plot presenting pooled risk ratio with 95% confidence interval (CI) for cancer reoccurrence among cancer survivors in cohort studies.

ESM Figure 8. Forest plot presenting pooled risk ratio with 95% confidence interval (CI) for cancer mortality among cancer survivors in cohort studies.

ESM Figure 9. Forest plot presenting pooled risk ratios with 95% confidence interval (CI) for cancer mortality in cohort studies and RCTs.

ESM Figure 10. Forest plot presenting pooled odds and risk ratios with 95% confidence interval (CI) for colorectal cancer in case-control and cohort studies.

ESM Figure 11. Forest plot presenting pooled odds and risk ratios with 95% confidence interval (CI) for endometrial cancer in case-control and cohort studies.

ESM Figure 12. Forest plot presenting pooled odds and risk ratios with 95% confidence interval (CI) for esophageal cancer in case-control and cohort studies.

ESM Figure 13. Forest plot presenting pooled risk ratio with 95% confidence interval (CI) for gallbladder cancer in cohort studies.

ESM Figure 14. Forest plot presenting pooled odds and risk ratios with 95% confidence interval (CI) for gastric cancer in case-control and cohort studies.

ESM Figure 15. Forest plot presenting pooled risk ratio with 95% confidence interval (CI) for glioma in cohort studies.

ESM Figure 16. Forest plot presenting pooled odds and risk ratios with 95% confidence interval (CI) for head and neck cancer in case-control and cohort studies.

ESM Figure 17. Forest plot presenting pooled odds and risk ratios with 95% confidence interval (CI) for liver cancer in case-control and cohort studies.

ESM Figure 18. Forest plot presenting pooled risk ratio with 95% confidence interval (CI) for ovarian cancer in cohort studies.

ESM Figure 19. Forest plot presenting pooled odds and risk ratios with 95% confidence interval (CI) for pancreatic cancer in case-control and cohort studies.

ESM Figure 20. Forest plot presenting pooled odds and risk ratios with 95% confidence interval (CI) for prostate cancer in case-control and cohort studies.

ESM Figure 21. Forest plot presenting pooled risk ratio with 95% confidence interval (CI) for respiratory cancer in cohort studies.

ESM Figure 22. Forest plot presenting pooled risk ratio with 95% confidence interval (CI) for skin cancer in cohort studies.

ESM Figure 23. Funnel plot showing study precision against the relative risk effect estimate for cancer mortality.

ESM Figure 24. Funnel plot showing study precision against the relative risk effect estimate for breast cancer.

ESM Figure 25. Funnel plot showing study precision against the relative risk effect estimate for colorectal cancer.

ESM Table 2. Subgroup analysis for association between adherence to the MedDiet and risk of cancer mortality.

ESM Table 3. Subgroup analysis for association between adherence to the MedDiet and risk of breast cancer.

ESM Table 4. Subgroup analysis for association between adherence to the MedDiet and risk of colorectal cancer.

ESM Table 5. Item-level scoring for NutriGrade tool and quality of evidence for identified associations in prospective observational studies and randomized controlled trials.

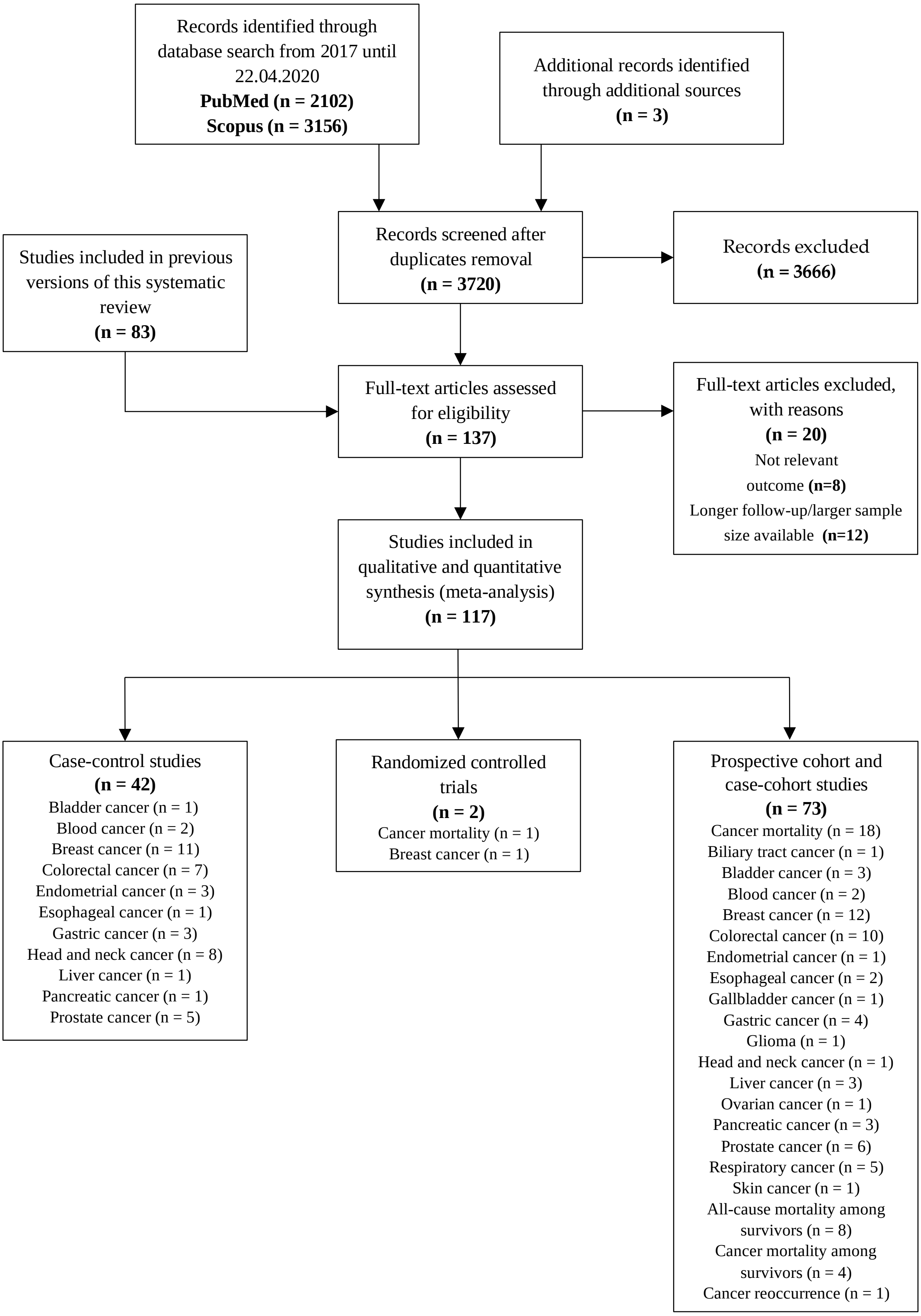

ESM Figure 1. Flowchart of study selection process in the updated systematic review.

ESM Table 1. Reasons for study exclusion at the full-text eligibility assessment step.

[1-8]	Not relevant outcome
[9-20]	Longer follow-up/larger sample size available

References:

1. Alvarez-Alvarez I, Zazpe I, Pérez de Rojas J, Bes-Rastrollo M, Ruiz-Canela M, Fernandez-Montero A, Hidalgo-Santamaría M, Martínez-González MA (2018) Mediterranean diet, physical activity and their combined effect on all-cause mortality: The Seguimiento Universidad de Navarra (SUN) cohort. Preventive Medicine 106:45-52. doi:10.1016/j.ypmed.2017.09.021
2. Bauer SR, Van Blarigan EL, Stampfer MJ, Chan JM, Kenfield SA (2018) Mediterranean diet after prostate cancer diagnosis and urinary and sexual functioning: The health professionals follow-up study. The Prostate 78 (3):202-212. doi:10.1002/pros.23457
3. Fasanelli F, Giraudo MT, Vineis P, Fiano V, Fiorito G, Grasso C, Polidoro S, Trevisan M, Grioni S, Krogh V, Mattiello A, Panico S, Giurdanella MC, Tumino R, De Marco L, Ricceri F, Sacerdote C (2019) DNA methylation, colon cancer and Mediterranean diet: results from the EPIC-Italy cohort. Epigenetics 14 (10):977-988. doi:10.1080/15592294.2019.1629230
4. Ghanavti M, Movahed M, Rashidkhani B, Rakhsha A, Hejazi E (2019) Index-Based Dietary Patterns and the Risk of Prostate Cancer among Iranian Men. Asian Pacific Journal of Cancer Prevention 20 (5):1393-1401. doi:10.31557/apjcp.2019.20.5.1393
5. Krusińska B, Hawrysz I, Słowińska MA, Wądołowska L, Biernacki M, Czerwińska A, Gołota JJ (2017) Dietary patterns and breast or lung cancer risk: A pooled analysis of 2 case-control studies in north-eastern Poland. Advances in Clinical and Experimental Medicine 26 (9):1367-1375. doi:10.17219/acem/65433
6. Shikany JM, Safford MM, Bryan J, Newby PK, Richman JS, Durant RW, Brown TM, Judd SE (2018) Dietary Patterns and Mediterranean Diet Score and Hazard of Recurrent Coronary Heart Disease Events and All-Cause Mortality in the REGARDS Study. Journal of the American Heart Association 7 (14). doi:10.1161/jaha.117.008078
7. Urquiza-Salvat N, Pascual-Geler M, Lopez-Guarnido O, Rodrigo L, Martinez-Burgos A, Cozar JM, Ocaña-Peinado FM, Álvarez-Cubero MJ, Rivas A (2019) Adherence to Mediterranean diet and risk of prostate cancer. The Aging Male 22 (2):102-108. doi:10.1080/13685538.2018.1450854
8. Zaslavsky O, Zelber-Sagi S, Shikany JM, Orchard T, Wallace R, Snetselaar L, Tinker L (2018) Anatomy of the Mediterranean Diet and Mortality Among Older Women with Frailty. Journal of nutrition in gerontology and geriatrics 37 (3-4):269-281. doi:10.1080/21551197.2018.1496217
9. Bonaccio M, Di Castelnuovo A, Costanzo S, De Curtis A, Persichillo M, Cerletti C, Donati MB, de Gaetano G, Iacoviello L (2019) Impact of combined healthy lifestyle factors on survival in an adult general population and in high-risk groups: prospective results from the Moli-sani Study. Journal of Internal Medicine 286 (2):207-220. doi:10.1111/joim.12907
10. Buckland G, Agudo A, Travier N, Huerta JM, Cirera L, Tormo MJ, Navarro C, Chirlaque MD, Moreno-Iribas C, Ardanaz E, Barricarte A, Etxeberria J, Marin P, Quiros JR, Redondo ML, Larranaga N, Amiano P, Dorronsoro M, Arriola L, Basterretxea M, Sanchez MJ, Molina E, Gonzalez CA (2011) Adherence to the Mediterranean diet reduces mortality in the Spanish cohort of the European Prospective Investigation into Cancer and Nutrition (EPIC-Spain). The British Journal of Nutrition 106 (10):1581-1591. doi:10.1017/s0007114511002078
11. Buckland G, Ros MM, Roswall N, Bueno-de-Mesquita HB, Travier N, Tjonneland A, Kiemeney LA, Sacerdote C, Tumino R, Ljungberg B, Gram IT, Weiderpass E, Skeie G, Malm J, Ehrnstrom R, Chang-Claude J, Mattiello A, Agnoli C, Peeters PH, Boutron-Ruault MC, Fagherazzi G, Clavel-Chapelon F, Nilsson LM, Amiano P, Trichopoulou A, Oikonomou E, Tsiotas K, Sanchez MJ, Overvad K, Quiros JR, Chirlaque MD, Barricarte A, Key TJ, Allen NE, Khaw KT, Wareham N, Riboli E, Kaaks R, Boeing H, Palli D, Romieu I, Romaguera D, Gonzalez CA (2014) Adherence to the Mediterranean diet and risk of bladder cancer in the EPIC cohort study. International Journal of Cancer 134 (10):2504-2511. doi:10.1002/ijc.28573
12. Couto E, Boffetta P, Lagiou P, Ferrari P, Buckland G, Overvad K, Dahm CC, Tjonneland A, Olsen A, Clavel-Chapelon F, Boutron-Ruault MC, Cottet V, Trichopoulos D, Naska A, Benetou V, Kaaks R, Rohrmann S, Boeing H, von Ruesten A, Panico S, Pala V, Vineis P, Palli D, Tumino R, May A, Peeters PH, Bueno-de-Mesquita HB, Buchner FL, Lund E, Skeie G, Engeset D, Gonzalez CA, Navarro C, Rodriguez L, Sanchez MJ, Amiano P, Barricarte A, Hallmans G, Johansson I, Manjer J, Wirfart E, Allen NE, Crowe F, Khaw KT, Wareham N, Moskal A, Slimani N, Jenab M, Romaguera D, Mouw T, Norat T, Riboli E, Trichopoulou A (2011) Mediterranean dietary pattern and cancer risk in the EPIC cohort. British Journal of Cancer 104 (9):1493-1499. doi:10.1038/bjc.2011.106

13. Fasanelli F, Zugna D, Giraudo MT, Krogh V, Grioni S, Panico S, Mattiello A, Masala G, Caini S, Tumino R, Frasca G, Sciannameo V, Ricceri F, Sacerdote C (2017) Abdominal adiposity is not a mediator of the protective effect of Mediterranean diet on colorectal cancer. International Journal of Cancer 140 (10):2265-2271. doi:10.1002/ijc.30653
14. Fung TT, Hu FB, Wu K, Chiuve SE, Fuchs CS, Giovannucci E (2010) The Mediterranean and Dietary Approaches to Stop Hypertension (DASH) diets and colorectal cancer. American Journal of Clinical Nutrition 92 (6):1429-1435. doi:10.3945/ajcn.2010.29242
15. Galbete C, Kröger J, Jannasch F, Iqbal K, Schwingshackl L, Schwedhelm C, Weikert C, Boeing H, Schulze MB (2018) Nordic diet, Mediterranean diet, and the risk of chronic diseases: the EPIC-Potsdam study. BMC Medicine 16 (1):99. doi:10.1186/s12916-018-1082-y
16. Gnagnarella P, Maisonneuve P, Bellomi M, Rampinelli C, Bertolotti R, Spaggiari L, Palli D, Veronesi G (2013) Red meat, Mediterranean diet and lung cancer risk among heavy smokers in the COSMOS screening study. Annals of Oncology 24 (10):2606-2611. doi:10.1093/annonc/mdt302
17. Haridass V (2015) Diet Quality Scores and Risk of Incident Breast Cancer in the California Teachers Study 2015.
18. Hirko KA, Willett WC, Hankinson SE, Rosner BA, Beck AH, Tamimi RM, Eliassen AH (2016) Healthy dietary patterns and risk of breast cancer by molecular subtype. Breast Cancer Research and Treatment 155 (3):579-588. doi:10.1007/s10549-016-3706-2
19. Lopez-Garcia E, Rodriguez-Artalejo F, Li TY, Fung TT, Li S, Willett WC, Rimm EB, Hu FB (2014) The Mediterranean-style dietary pattern and mortality among men and women with cardiovascular disease. American Journal of Clinical Nutrition 99 (1):172-180. doi:10.3945/ajcn.113.068106
20. Toledo E, Salas-Salvado J, Donat-Vargas C, Buil-Cosiales P, Estruch R, Ros E, Corella D, Fito M, Hu FB, Aros F, Gomez-Gracia E, Romaguera D, Ortega-Calvo M, Serra-Majem L, Pinto X, Schroder H, Basora J, Sorli JV, Bullo M, Serra-Mir M, Martinez-Gonzalez MA (2015) Mediterranean Diet and Invasive Breast Cancer Risk Among Women at High Cardiovascular Risk in the PREDIMED Trial: A Randomized Clinical Trial. JAMA Internal Medicine 175 (11):1752-1760. doi:10.1001/jamainternmed.2015.4838

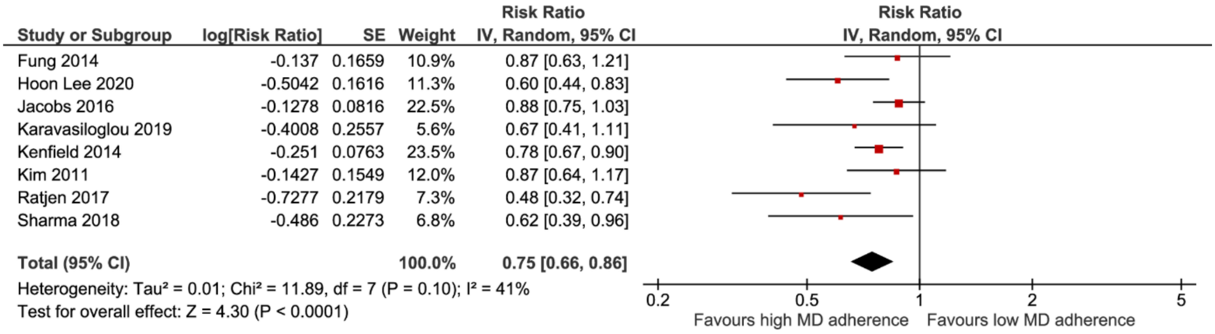

ESM Figure 2. Forest plot presenting pooled risk ratio with 95% confidence interval (CI) for all-cause mortality among cancer survivors in cohort studies. MD – Mediterranean diet;

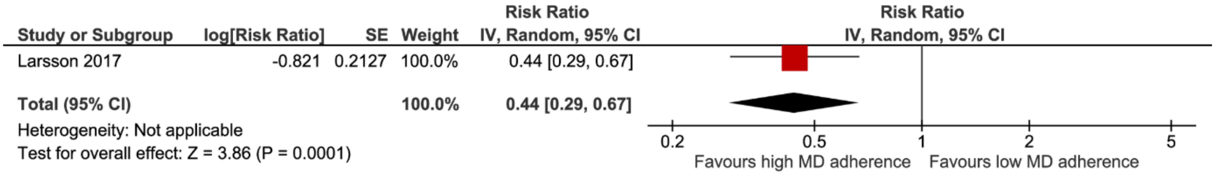

ESM Figure 3. Forest plot presenting pooled risk ratio with 95% confidence interval (CI) for biliary tract cancer in cohort studies. MD – Mediterranean diet;

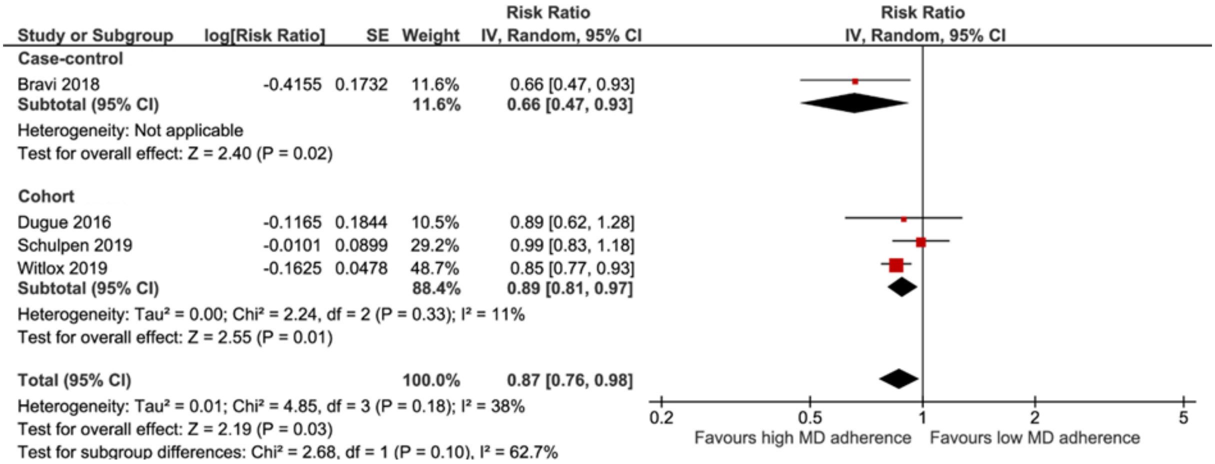

ESM Figure 4. Forest plot presenting pooled odds and risk ratios with 95% confidence interval (CI) for bladder cancer in case-control and cohort studies. MD – Mediterranean diet;

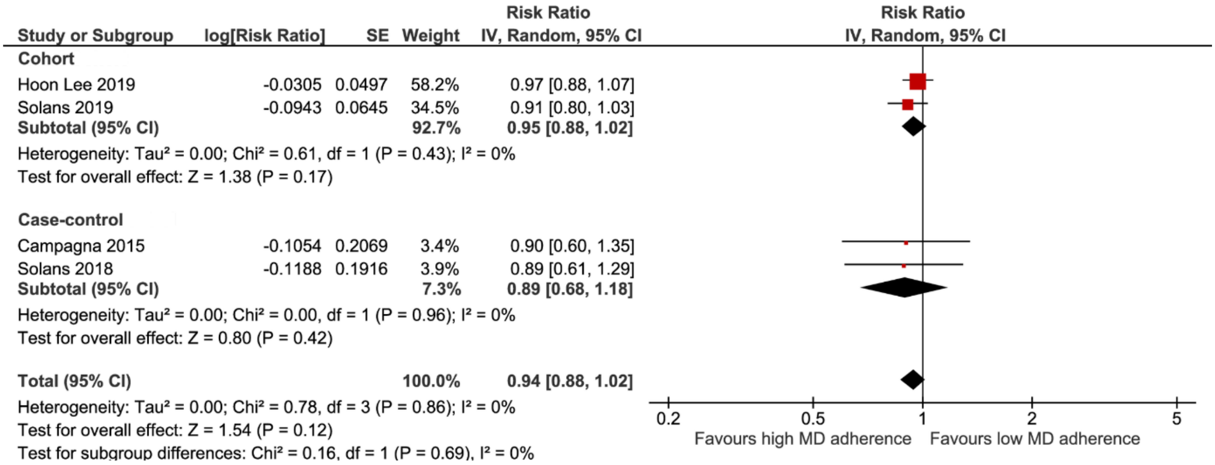

ESM Figure 5. Forest plot presenting pooled odds and risk ratios with 95% confidence interval (CI) for blood cancer in case-control and cohort studies. MD – Mediterranean diet;

Study or Subgroup	log[Risk Ratio]	SE	Weight	Risk Ratio IV, Random, 95% CI
**Case-control**				
Bessaoud 2012	-0.0305	0.2202	1.9%	0.97 [0.63, 1.49]
Castello 2014	-0.3011	0.2426	1.6%	0.74 [0.46, 1.19]
Castello 2017	-0.1054	0.1356	4.9%	0.90 [0.69, 1.17]
Demetriou 2012	-0.0101	0.1769	2.9%	0.99 [0.70, 1.40]
Krusinska 2018	-0.6616	0.3697	0.7%	0.52 [0.25, 1.07]
Mourouti 2014	-0.0943	0.0288	58.1%	0.91 [0.86, 0.96]
Murtaugh 2008	-0.2744	0.0957	9.5%	0.76 [0.63, 0.92]
Nkondjock 2007	-0.6162	0.5897	0.3%	0.54 [0.17, 1.72]
Pot 2014	0.0392	0.202	2.3%	1.04 [0.70, 1.55]
Turati 2018	-0.1985	0.0735	15.3%	0.82 [0.71, 0.95]
Wu 2009	-0.3567	0.1925	2.5%	0.70 [0.48, 1.02]
**Subtotal (95% CI)**			**100.0%**	**0.87 [0.82, 0.93]**
Heterogeneity: Tau² = 0.00; Chi² = 10.63, df = 10 (P = 0.39); I² = 6%				
Test for overall effect: Z = 4.43 (P < 0.00001)				
**Cohort**				
Boden 2019	-0.0202	0.035	21.9%	0.98 [0.92, 1.05]
Buckland 2013	-0.0619	0.0336	23.7%	0.94 [0.88, 1.00]
Butler 2010	-0.0408	0.1192	1.9%	0.96 [0.76, 1.21]
Cade 2011	-0.0408	0.1612	1.0%	0.96 [0.70, 1.32]
Couto 2013	0.3507	0.184	0.8%	1.42 [0.99, 2.04]
Dela Cruz 2020	0.01	0.0366	20.0%	1.01 [0.94, 1.09]
Fung 2006	-0.0202	0.0549	8.9%	0.98 [0.88, 1.09]
Gardeazabal 2020	-0.4463	0.3866	0.2%	0.64 [0.30, 1.37]
Haridass 2018	-0.0305	0.0439	13.9%	0.97 [0.89, 1.06]
Lavalette 2018	0.1222	0.1543	1.1%	1.13 [0.84, 1.53]
Petimar 2019	-0.1165	0.0839	3.8%	0.89 [0.76, 1.05]
van den Brandt 2017	-0.1393	0.0966	2.9%	0.87 [0.72, 1.05]
**Subtotal (95% CI)**			**100.0%**	**0.97 [0.94, 1.00]**
Heterogeneity: Tau² = 0.00; Chi² = 10.97, df = 11 (P = 0.45); I² = 0%				
Test for overall effect: Z = 1.80 (P = 0.07)				
**Total (95% CI)**			**100.0%**	**0.94 [0.90, 0.97]**
Heterogeneity: Tau² = 0.00; Chi² = 31.80, df = 22 (P = 0.08); I² = 31%				
Test for overall effect: Z = 3.24 (P = 0.001)				
Test for subgroup differences: Chi² = 9.41, df = 1 (P = 0.002), I² = 89.4%				
**RCT**				
Toledo 2018	-0.8989	0.3887	100.0%	0.41 [0.19, 0.87]
**Subtotal (95% CI)**			**100.0%**	**0.41 [0.19, 0.87]**
Heterogeneity: Not applicable				
Test for overall effect: Z = 2.31 (P = 0.02)				

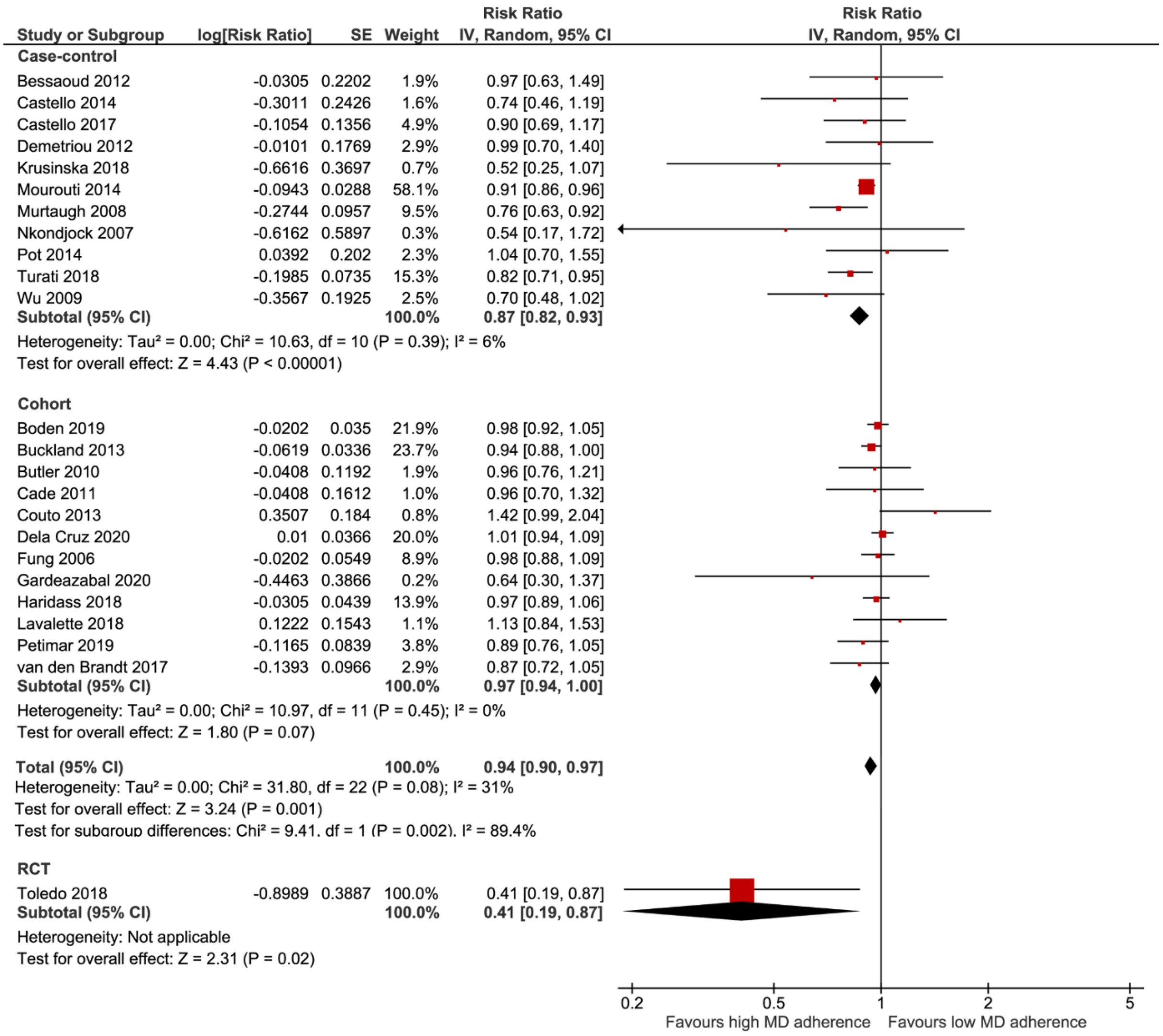

ESM Figure 6. Forest plot presenting pooled odds and risk ratios with 95% confidence interval (CI) for breast cancer in case-control, cohort studies and RCTs. MD – Mediterranean diet; RCT – Randomized controlled trial;

Study or Subgroup	log[Risk Ratio]	SE	Weight	Risk Ratio IV, Random, 95% CI
Cottet 2005	-0.4943	0.623	100.0%	0.61 [0.18, 2.07]
**Total (95% CI)**			**100.0%**	**0.61 [0.18, 2.07]**
Heterogeneity: Not applicable				
Test for overall effect: Z = 0.79 (P = 0.43)				

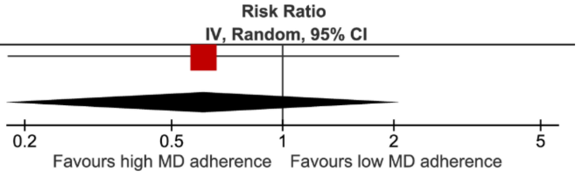

ESM Figure 7. Forest plot presenting pooled risk ratio with 95% confidence interval (CI) for cancer reoccurrence among cancer survivors in cohort studies. MD – Mediterranean diet;

Study or Subgroup	log[Risk Ratio]	SE	Weight	Risk Ratio IV, Random, 95% CI
Fung 2014	-0.1708	0.2665	8.5%	0.84 [0.50, 1.42]
Jacobs 2016	-0.0943	0.1055	54.1%	0.91 [0.74, 1.12]
Kenfield 2014	0.0139	0.1539	25.4%	1.01 [0.75, 1.37]
Kim 2011	0.1363	0.2232	12.1%	1.15 [0.74, 1.77]
**Total (95% CI)**			**100.0%**	**0.96 [0.82, 1.11]**
Heterogeneity: Tau² = 0.00; Chi² = 1.25, df = 3 (P = 0.74); I² = 0%				
Test for overall effect: Z = 0.59 (P = 0.56)				

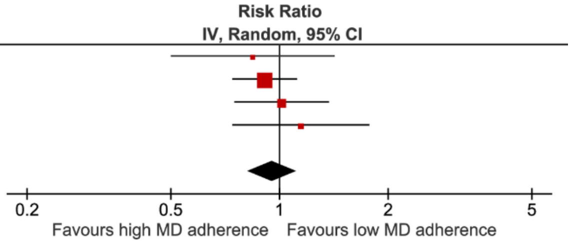

ESM Figure 8. Forest plot presenting pooled risk ratio with 95% confidence interval (CI) for cancer mortality among cancer survivors in cohort studies. MD – Mediterranean diet;

Study or Subgroup	log[Risk Ratio]	SE	Weight	Risk Ratio IV, Random, 95% CI
**Cohort**				
Bonaccio 2018	-0.1393	0.214	1.5%	0.87 [0.57, 1.32]
Cheng 2018	-0.0726	0.0519	7.2%	0.93 [0.84, 1.03]
Cuenca-García 2014	0.4886	0.2974	0.8%	1.63 [0.91, 2.92]
George 2014	-0.2231	0.0681	6.1%	0.80 [0.70, 0.91]
Harmon 2015	-0.1985	0.0321	8.5%	0.82 [0.77, 0.87]
Hashemian 2019	-0.462	0.1551	2.4%	0.63 [0.46, 0.85]
Knoops 2004	-0.1054	0.1282	3.2%	0.90 [0.70, 1.16]
Lagiou 2006	-0.2231	0.1729	2.1%	0.80 [0.57, 1.12]
Lassale 2016	-0.1985	0.0321	8.5%	0.82 [0.77, 0.87]
Martinez-Gonzalez 2012	0.0296	0.1757	2.0%	1.03 [0.73, 1.45]
Menotti 2012	-0.1744	0.0647	6.3%	0.84 [0.74, 0.95]
Neelakantan 2018	-0.1278	0.0486	7.4%	0.88 [0.80, 0.97]
Reedy 2014	-0.2231	0.0195	9.1%	0.80 [0.77, 0.83]
Sotos-Prieto 2017	-0.0202	0.0267	8.8%	0.98 [0.93, 1.03]
Tognon 2012	-0.0513	0.0219	9.0%	0.95 [0.91, 0.99]
Vormund 2015	-0.0305	0.0215	9.0%	0.97 [0.93, 1.01]
Warensjö Lemming 2018	-0.2107	0.0782	5.5%	0.81 [0.69, 0.94]
Whalen 2017	-0.4463	0.1468	2.6%	0.64 [0.48, 0.85]
**Subtotal (95% CI)**			**100.0%**	**0.87 [0.82, 0.92]**
Heterogeneity: Tau² = 0.01; Chi² = 101.35, df = 17 (P < 0.00001); I² = 83%				
Test for overall effect: Z = 5.07 (P < 0.00001)				
**RCT**				
de Lorgeril 1998	-0.2844	0.7594	100.0%	0.75 [0.17, 3.33]
**Subtotal (95% CI)**			**100.0%**	**0.75 [0.17, 3.33]**
Heterogeneity: Not applicable				
Test for overall effect: Z = 0.37 (P = 0.71)				
Test for subgroup differences: Chi² = 0.03, df = 1 (P = 0.85), I² = 0%				

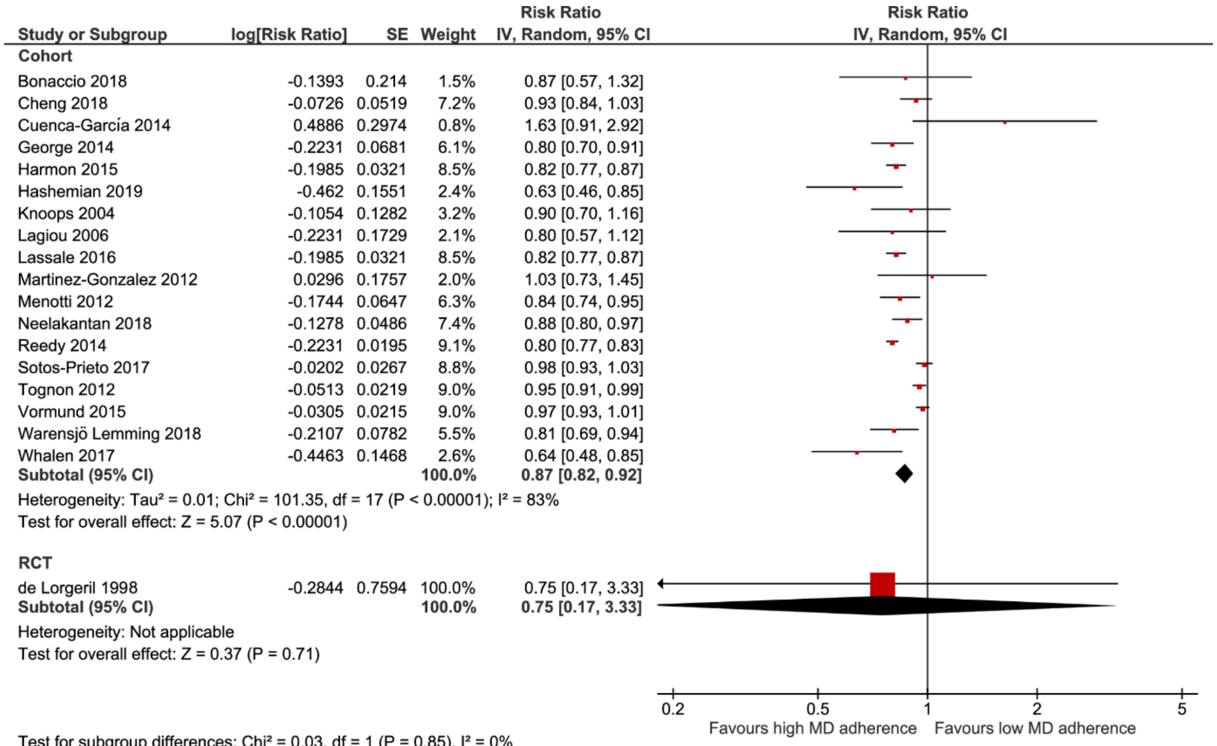

ESM Figure 9. Forest plot presenting pooled risk ratios with 95% confidence interval (CI) for cancer mortality in cohort studies and RCTs. MD – Mediterranean diet; RCT – Randomized controlled trial;

Study or Subgroup	log[Risk Ratio]	SE	Weight	Risk Ratio IV, Random, 95% CI
**Case-control**				
Castello 2019	-0.4308	0.1041	6.0%	0.65 [0.53, 0.80]
Dixon 2007	-0.1508	0.0631	7.7%	0.86 [0.76, 0.97]
Grosso 2014	-0.7765	0.2533	2.2%	0.46 [0.28, 0.76]
Kontou 2012	-0.1278	0.0237	9.0%	0.88 [0.84, 0.92]
Nasab 2019	-1.682	0.3704	1.2%	0.19 [0.09, 0.38]
Rosato 2016	-0.6539	0.097	6.3%	0.52 [0.43, 0.63]
Whalen 2014	-0.2614	0.1906	3.3%	0.77 [0.53, 1.12]
**Subtotal (95% CI)**			**35.5%**	**0.64 [0.52, 0.79]**
Heterogeneity: Tau² = 0.06; Chi² = 56.90, df = 6 (P < 0.00001); I² = 89%				
Test for overall effect: Z = 4.09 (P < 0.0001)				
**Cohort**				
Bamia 2013	-0.1165	0.0544	8.0%	0.89 [0.80, 0.99]
Boden 2019	0.0198	0.04	8.5%	1.02 [0.94, 1.10]
Cheng 2018	0.01	0.0808	6.9%	1.01 [0.86, 1.18]
Jones 2017	-0.1985	0.1812	3.5%	0.82 [0.57, 1.17]
Lavalette 2018	0.0198	0.3537	1.3%	1.02 [0.51, 2.04]
Park 2017	-0.1165	0.0544	8.0%	0.89 [0.80, 0.99]
Petimar 2019	-0.0943	0.0722	7.3%	0.91 [0.79, 1.05]
Schulpen 2019	0.0296	0.0745	7.2%	1.03 [0.89, 1.19]
Torres Stone 2017	-0.2357	0.058	7.9%	0.79 [0.71, 0.89]
Vargas 2016	-0.0943	0.1055	5.9%	0.91 [0.74, 1.12]
**Subtotal (95% CI)**			**64.5%**	**0.92 [0.87, 0.99]**
Heterogeneity: Tau² = 0.00; Chi² = 18.16, df = 9 (P = 0.03); I² = 50%				
Test for overall effect: Z = 2.39 (P = 0.02)				
**Total (95% CI)**			**100.0%**	**0.83 [0.76, 0.90]**
Heterogeneity: Tau² = 0.02; Chi² = 87.83, df = 16 (P < 0.00001); I² = 82%				
Test for overall effect: Z = 4.36 (P < 0.0001)				
Test for subgroup differences: Chi² = 10.36, df = 1 (P = 0.001), I² = 90.3%				

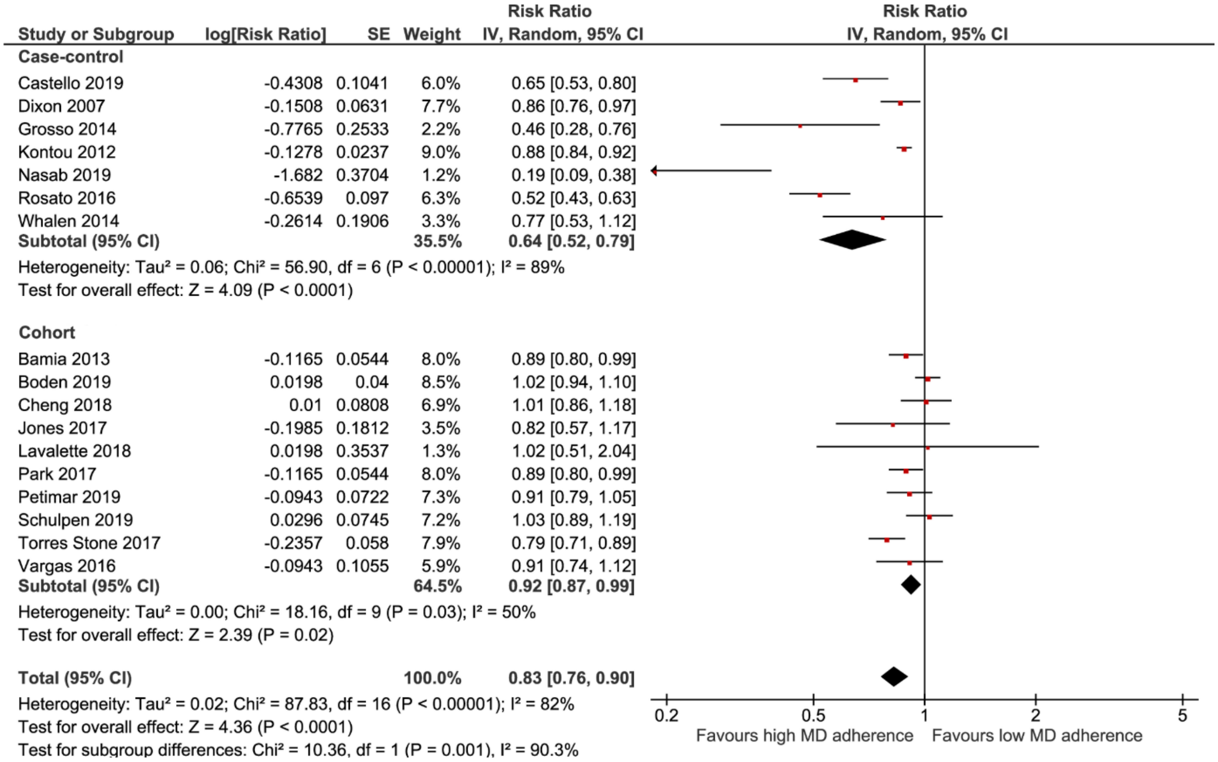

ESM Figure 10. Forest plot presenting pooled odds and risk ratios with 95% confidence interval (CI) for colorectal cancer in case-control and cohort studies. MD – Mediterranean diet;

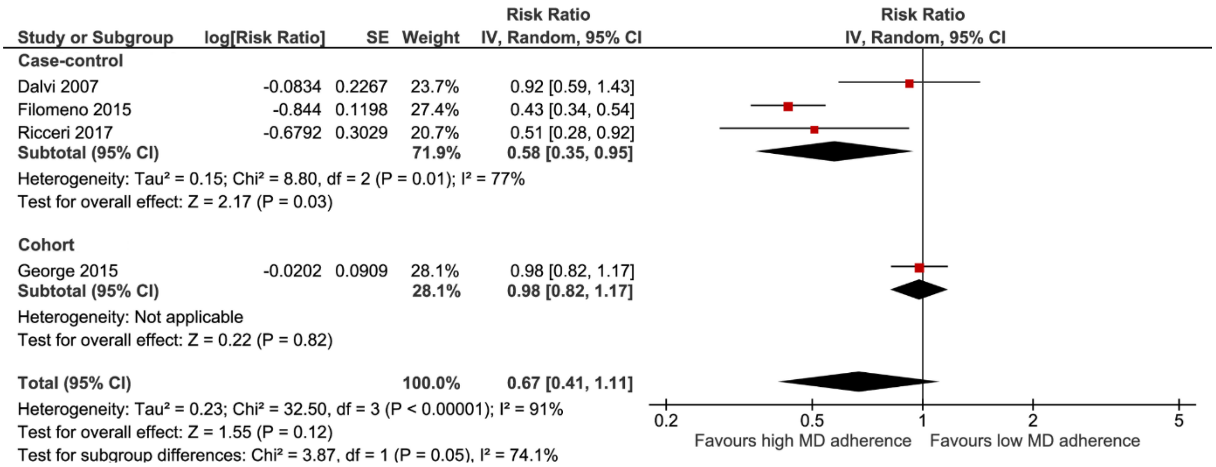

ESM Figure 11. Forest plot presenting pooled odds and risk ratios with 95% confidence interval (CI) for endometrial cancer in case-control and cohort studies. MD – Mediterranean diet;

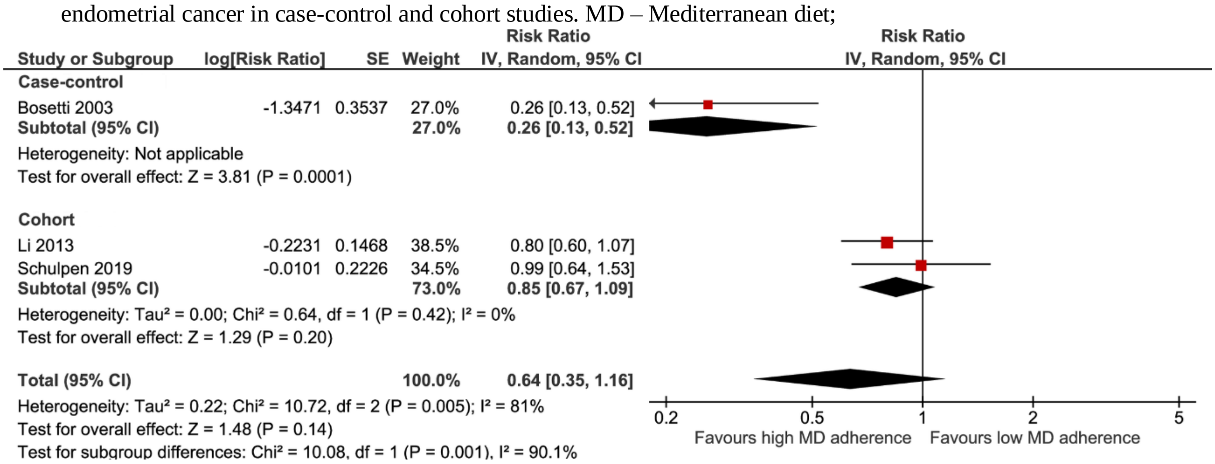

ESM Figure 12. Forest plot presenting pooled odds and risk ratios with 95% confidence interval (CI) for esophageal cancer in case-control and cohort studies. MD – Mediterranean diet;

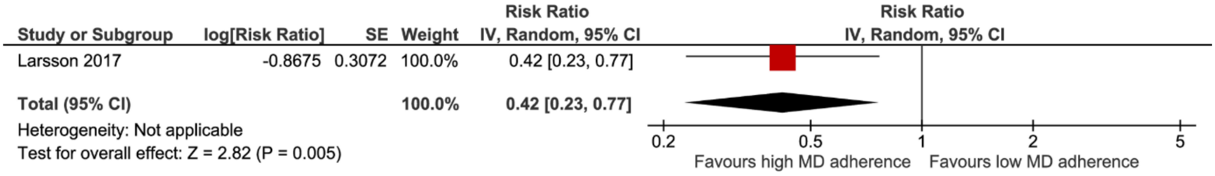

ESM Figure 13. Forest plot presenting pooled risk ratio with 95% confidence interval (CI) for gallbladder cancer in cohort studies. MD – Mediterranean diet;

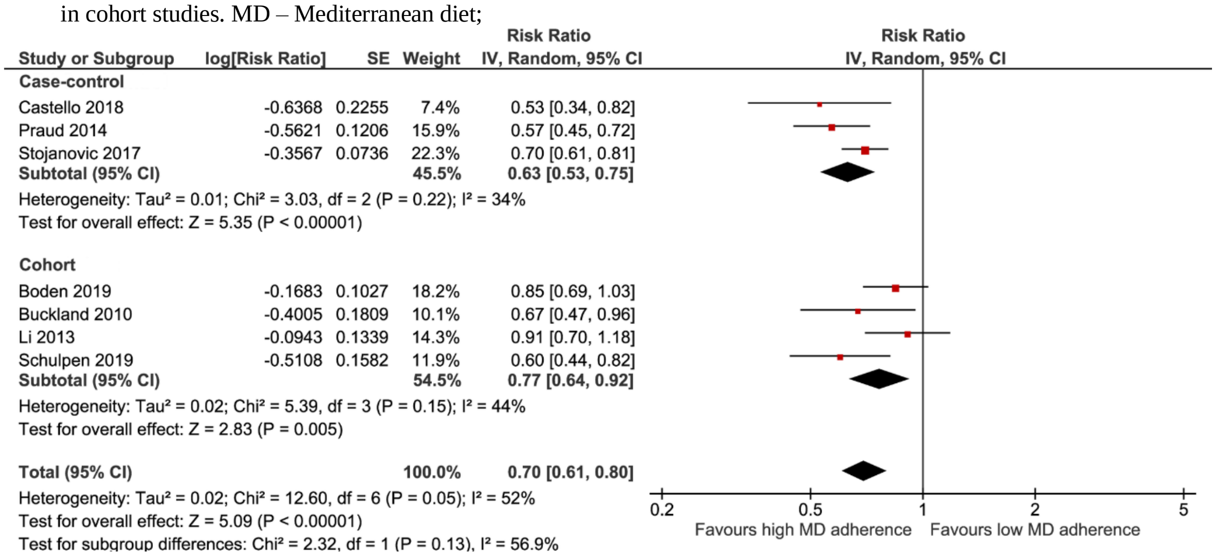

ESM Figure 14. Forest plot presenting pooled odds and risk ratios with 95% confidence interval (CI) for gastric cancer in case-control and cohort studies. MD – Mediterranean diet;

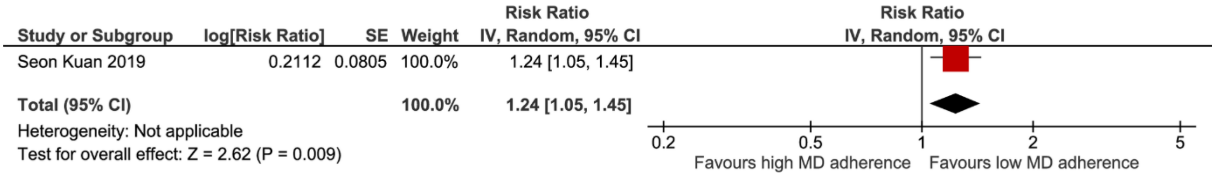

ESM Figure 15. Forest plot presenting pooled risk ratio with 95% confidence interval (CI) for glioma in cohort studies. MD – Mediterranean diet;

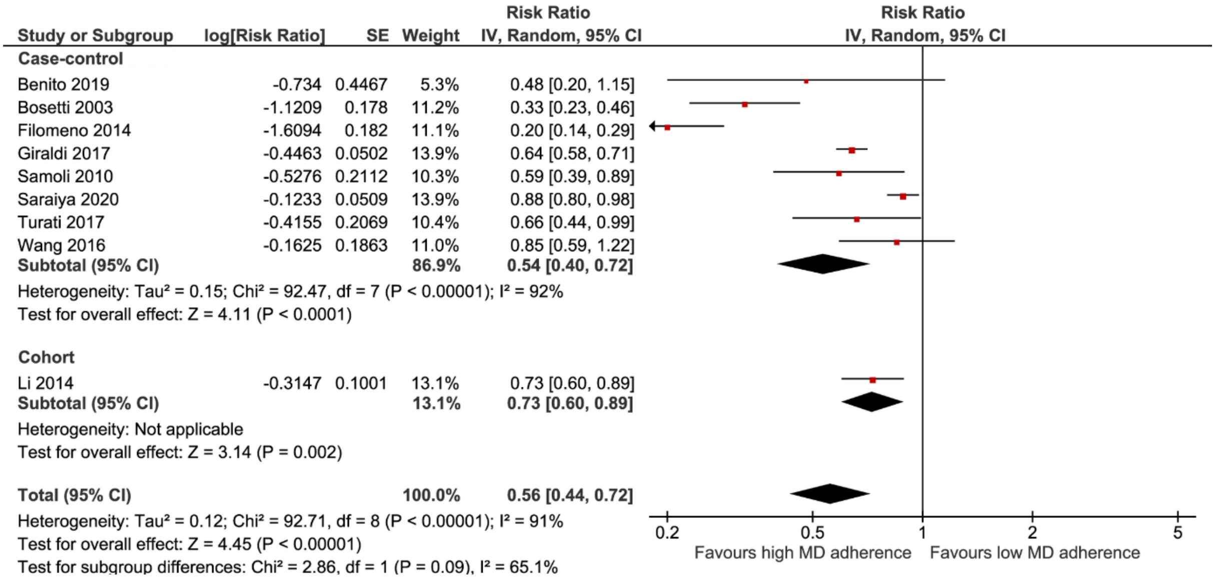

ESM Figure 16. Forest plot presenting pooled odds and risk ratios with 95% confidence interval (CI) for head and neck cancer in case-control and cohort studies. MD – Mediterranean diet;

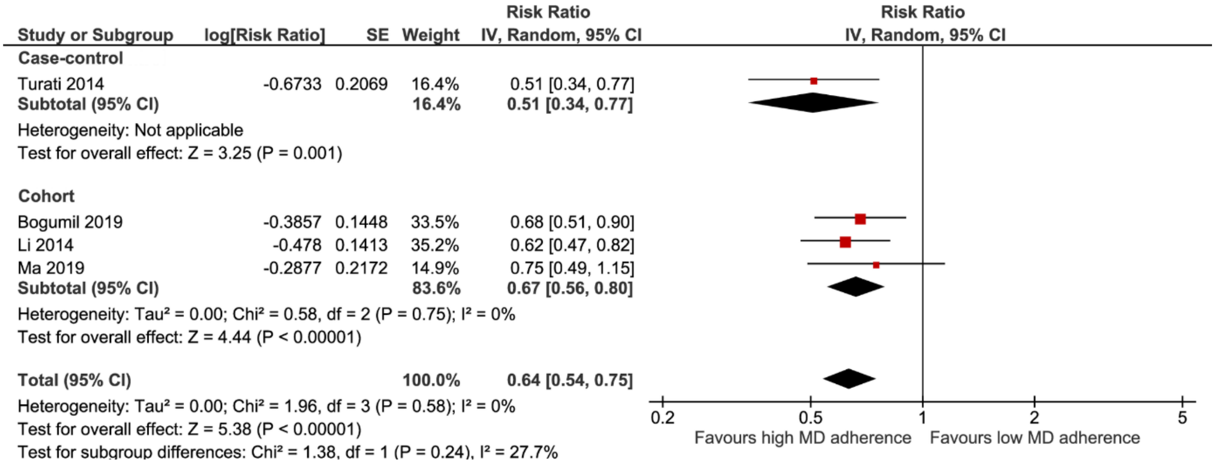

ESM Figure 17. Forest plot presenting pooled odds and risk ratios with 95% confidence interval (CI) for liver cancer in case-control and cohort studies. MD – Mediterranean diet;

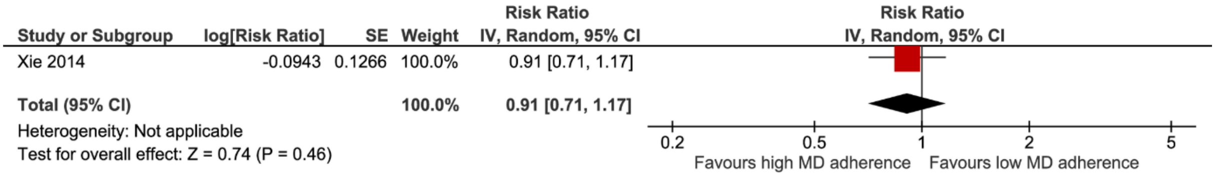

ESM Figure 18. Forest plot presenting pooled risk ratio with 95% confidence interval (CI) for ovarian cancer in cohort studies. MD – Mediterranean diet;

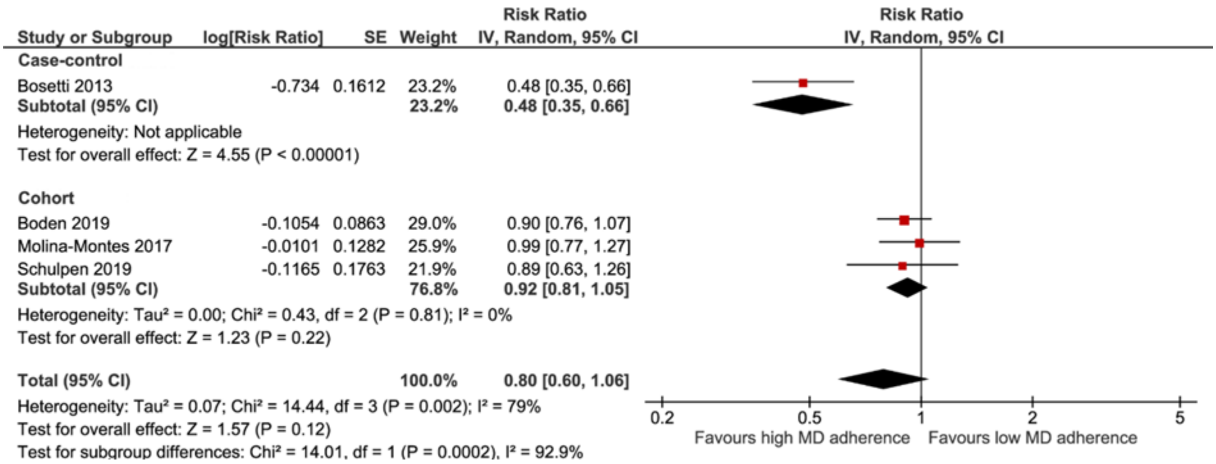

ESM Figure 19. Forest plot presenting pooled odds and risk ratios with 95% confidence interval (CI) for pancreatic cancer in case-control and cohort studies. MD – Mediterranean diet;

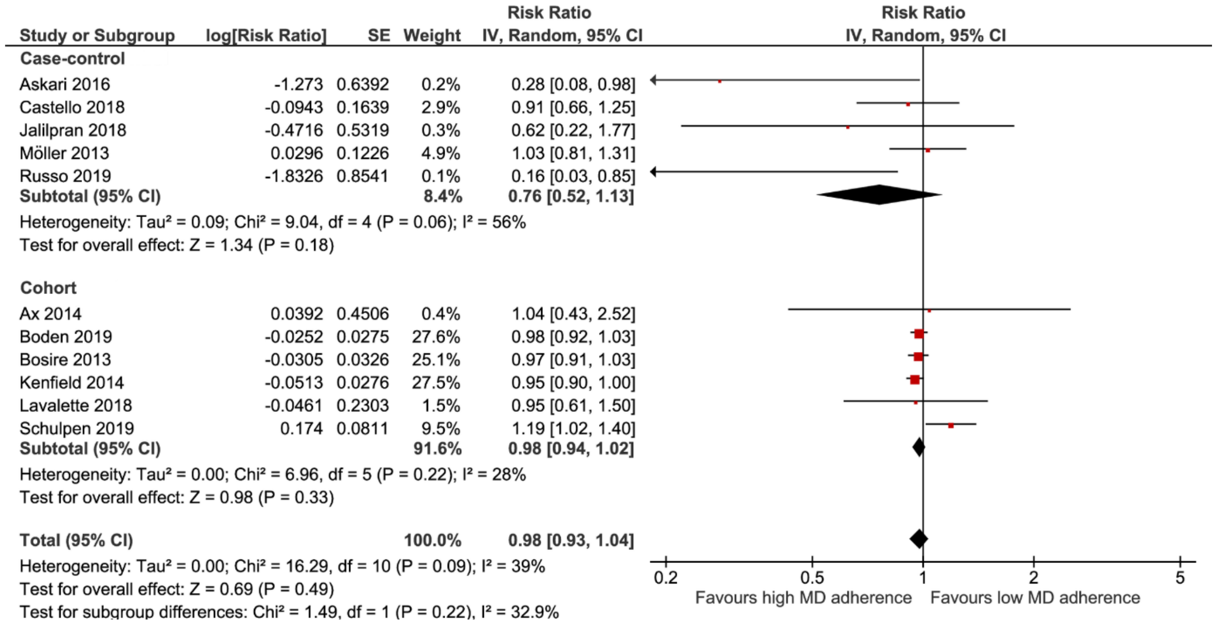

ESM Figure 20. Forest plot presenting pooled odds and risk ratios with 95% confidence interval (CI) for prostate cancer in case-control and cohort studies. MD – Mediterranean diet;

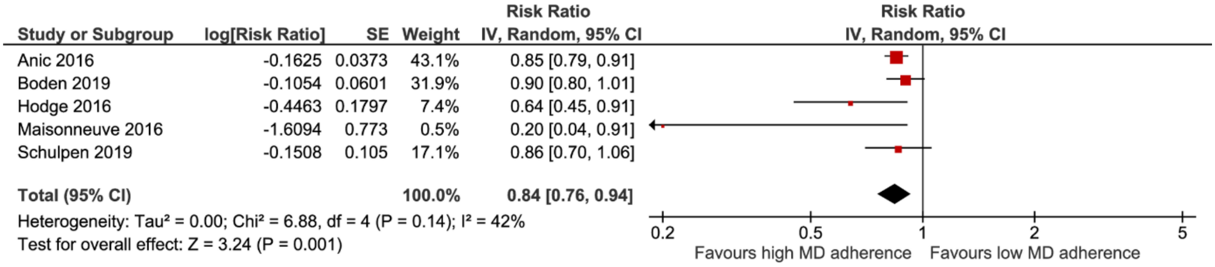

ESM Figure 21. Forest plot presenting pooled risk ratio with 95% confidence interval (CI) for respiratory cancer in cohort studies. MD – Mediterranean diet;

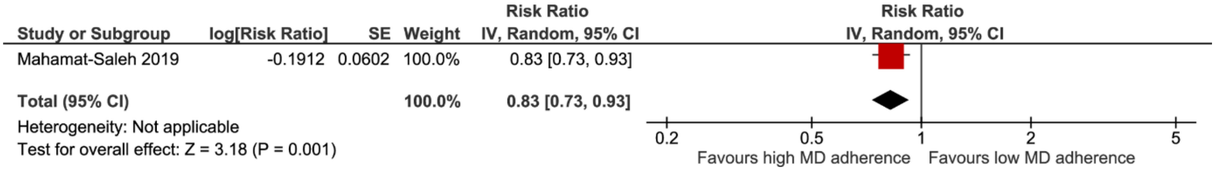

ESM Figure 22. Forest plot presenting pooled risk ratio with 95% confidence interval (CI) for skin cancer in cohort studies. MD – Mediterranean diet;

ESM Table 2. Subgroup analysis for association between adherence to the MedDiet and risk of cancer mortality.

Subgroup	*N*	RR	95%CI	I^2 (%)	$P_{subgroup}$
**Sex**					
Male	6	0.88	0.82, 0.95	90	0.80
Female	11	0.89	0.84, 0.95	77	
**MD score**					
tMedDiet	6	0.88	0.77, 0.99	77	0.69
aMedDiet	7	0.85	0.78, 0.93	87	
**Location**					
Mediterranean countries	4	0.85	0.76, 0.95	0	0.82
Non-Mediterranean countries	13	0.86	0.81, 0.92	87	

CI – Confidence interval; NA – Not applicable; aMedDiet – Fung MedDiet Score; tMedDiet – Trichopoulou MedDiet score;

ESM Table 3. Subgroup analysis for association between adherence to the MedDiet and risk of breast cancer.

Subgroup	*N*	RR	95%CI	I^2 (%)	$P_{subgroup}$
**MD score**					
tMedDiet	4	0.99	0.87, 1.13	38	0.82
aMedDiet	5	0.98	0.93, 1.02	0	
**Location**					
Mediterranean countries	2	0.95	0.57, 1.59	46	0.94
Non-Mediterranean countries	8	0.98	0.94, 1.02	0	
**Menopausal status**					
Premenopausal	5	0.99	0.71, 1.37	81	0.85
Postmenopausal	8	0.95	0.92, 0.99	0	
**Receptor expression**					
ER+	4	0.98	0.89, 1.06	30	0.85
ER-	4	0.86	0.64, 1.15	73	
PR+	2	1.00	0.91, 1.09	0	
PR-	2	0.91	0.61, 1.36	82	
ER+/PR+	4	0.95	0.88, 1.02	21	
ER-/PR-	4	0.89	0.66, 1.20	76	
ER+/PR-	1	0.98	0.81, 1.19	NA	
ER-/PR+	1	0.82	0.56, 1.20	NA	
ER-/PR-/HER2-	1	0.66	0.37, 1.19	NA	

CI – Confidence interval; NA – Not applicable; aMedDiet – Fung MedDiet Score; tMedDiet – Trichopoulou MedDiet score;

ESM Table 4. Subgroup analysis for association between adherence to the MedDiet and risk of colorectal cancer.

Subgroup	*N*	RR	95%CI	I^2 (%)	$P_{subgroup}$
**Sex**					
Male	6	0.88	0.78, 0.98	72	0.14
Female	9	0.91	0.90, 1.00	0	
**MD score**					
tMedDiet	2	0.88	0.80, 0.98	0	0.84
aMedDiet	5	0.89	0.82, 0.98	53	
**Location**					
Mediterranean countries	1	1.02	0.51, 2.04	NA	0.79
Non-Mediterranean countries	8	0.93	0.86, 1.00	61	
**Anatomical site**					
Proximal colon	5	1.01	0.93, 1.09	0	0.03
Distal colon	5	0.88	0.79, 0.96	0	
Rectum	7	0.86	0.75, 0.98	42	

CI – Confidence interval; NA – Not applicable; aMedDiet – Fung MedDiet Score; tMedDiet – Trichopoulou MedDiet score;

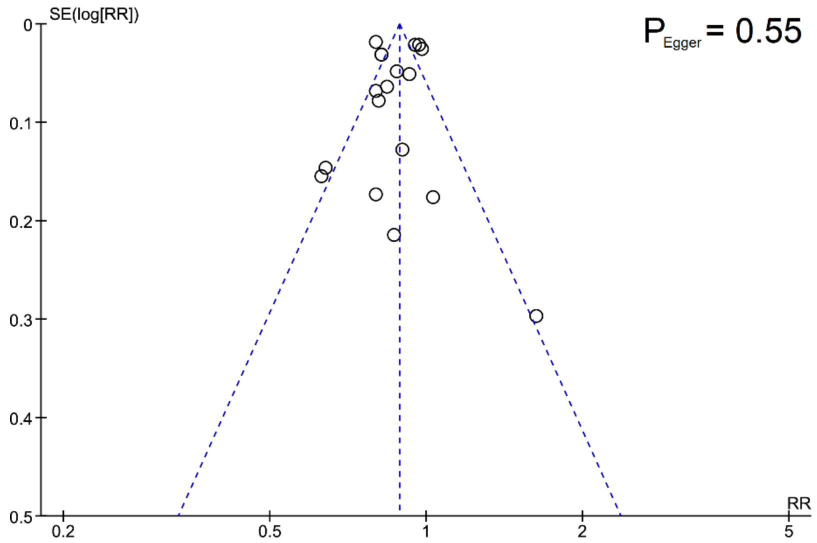

ESM Figure 23. Funnel plot showing study precision against the relative risk effect estimate for cancer mortality. P_{Egger} – P-value for Egger's linear regression test; SE – Standard error;

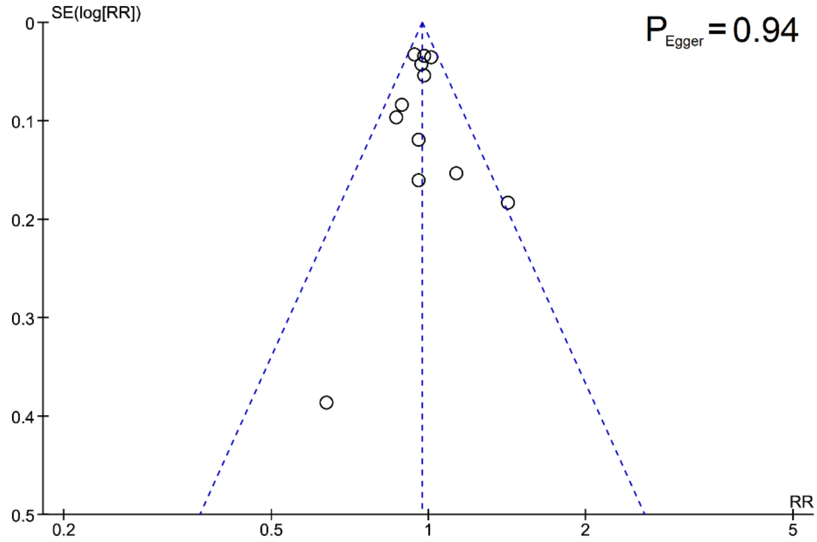

ESM Figure 24. Funnel plot showing study precision against the relative risk effect estimate for breast cancer. P_{Egger} – P-value for Egger's linear regression test; SE – Standard error;

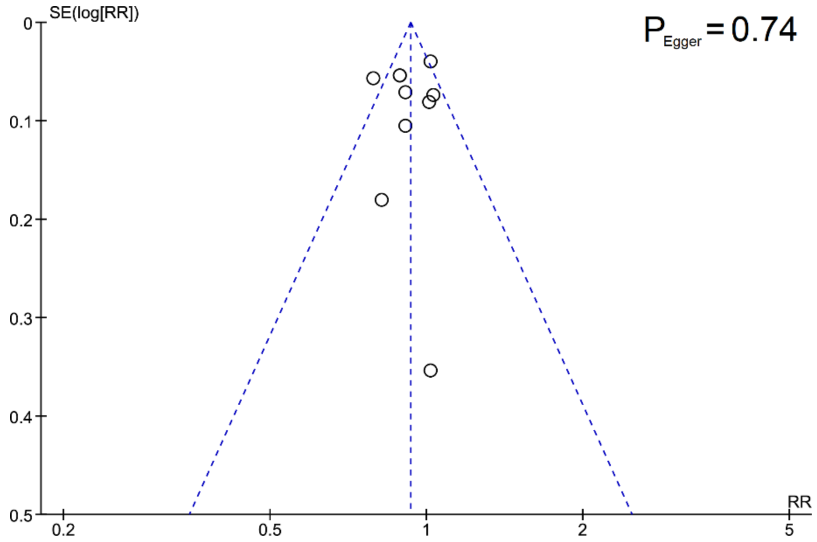

ESM Figure 25. Funnel plot showing study precision against the relative risk effect estimate for colorectal cancer. P_{Egger} – P-value for Egger's linear regression test; SE – Standard error;

ESM Table 5. Item-level scoring for NutriGrade tool and quality of evidence for identified associations in cohort studies and randomized controlled trials.

Outcome	Risk of bias, study quality, and study limitations	Precision	Heterogeneity	Directness	Publication bias	Funding bias	Effect size[1]	Dose response[1]	Total score	Quality of evidence
**Cohort studies**										
All-cause mortality among survivors	2 points	1 points	0.4 points	0 points	0 points	1 point	0.5 points	0 points	4.9 points	Low
Biliary tract cancer	1 point	0 points	0 points	0 points	0 points	1 point	0 points	0 points	2 points	Very low
Bladder cancer	2 points	1 point	0 point	1 point	0 points	1 point	0 points	0 points	5 points	Low
Blood cancer	2 points	1 point	0 point	0 points	0 points	1 point	0 points	0 points	4 points	Low
Breast cancer	2 points	1 points	0.8 points	0 points	0.5 points	1 point	0 points	0 points	5.3 points	Low
Cancer reoccurrence among survivors	1 point	0 points	0 points	0 points	0 points	1 point	0 points	0 points	2 points	Very low
Cancer mortality	2 points	1 point	0.6 points	1 point	0.5 points	1 point	0 points	0 points	6.1 points	Moderate
Cancer mortality among survivors	2 points	0 points	0 points	0 point	0 points	1 point	0 points	0 points	3 points	Very low
Colorectal cancer	2 points	1 point	0.6 points	1 point	1 point	1 point	0 points	0 points	6.6 points	Moderate
Endometrial cancer	1 point	0 points	0 points	0 points	0 points	1 point	0 points	0 points	2 points	Very low
Esophageal cancer	2 points	0 points	0 point	0 points	0 points	1 point	0 points	0 points	3 points	Very low
Gallbladder cancer	1 point	0 points	0 points	0 points	0 points	1 point	0 points	0 points	2 points	Very low
Gastric cancer	2 points	1 point	0 point	1 point	0 points	1 point	1 point	0 points	5 points	Very low
Glioma	1 point	1 point	0 points	0 points	0 points	1 point	0 points	0 points	3 points	Very low
Head and neck cancer	1 point	1 point	0 points	0 points	0 points	1 point	0 points	0 points	3 points	Low
Liver cancer	2 points	1 point	0 points	0 points	0 points	1 point	1 point	0 points	4 points	Very low
Ovarian cancer	1 point	0 points	0 points	0 points	0 points	1 point	0 points	0 points	2 points	Very low
Pancreatic cancer	2 points	0 points	0 points	0 points	0 points	1 point	0 points	0 points	3 points	Low
Prostate cancer	2 points	1 point	0.8 points	1 point	0 points	1 point	0 points	0 points	5.8 points	Very low
Respiratory cancer	2 points	1 point	0 points	1 point	0 points	1 point	0 points	0 points	5 points	Very low
Skin cancer	1 point	1 point	0 points	0 points	0 points	1 point	0 points	0 points	3 points	Low
**Randomized controlled trials**	**Risk of bias, study quality, and study limitations**	**Precision**	**Heterogeneity**	**Directness**	**Publication bias**	**Funding bias**	**Study design[2]**	-	**Total score**	Low
Cancer mortality	2.25 points	0 points	0 points	0 points	0 points	1 point	2 points	-	5.25 points	Very low
Breast cancer	2 points	0 points	0 points	0 points	0 points	1 point	2 points	-	5 points	Low

[1]Applies only for cohort studies; [2]Applies only for randomized controlled trials;
